# Triterpenoid Exposure and HO-1 Blockade Sensitize Neoplastic Mast Cells Against Midostaurin and Avapritinib

**DOI:** 10.3390/cells15201898

**Published:** 2026-10-19

**Authors:** Max Vincent John, Yüksel Filik Schüppler, Nadine Witzeneder, Mathias Schneeweiss-Gleixner, Barbara Peter, Daniela Berger, Gabriele Stefanzl, Isma Ishaq-Parveen, Jun Fang, Waliul Islam, Juliana Schwaab, Andreas Reiter, Marina Konopleva, Michael Andreeff, Gregor Hoermann, Michel Arock, Johannes Zuber, Klaus Schmetterer, Wolfgang R. Sperr, Peter Valent, Karoline V. Gleixner

**Affiliations:** 1 Department of Internal Medicine I, Division of Hematology and Hemostaseology, Medical University of Vienna, 1090 Vienna, Austria; max.john@meduniwien.ac.at (M.V.J.); yueksel.filik@lbg.ac.at (Y.F.S.); barbara.peter@lbg.ac.at (B.P.); daniela.berger@meduniwien.ac.at (D.B.); gabriele.stefanzl@meduniwien.ac.at (G.S.); isma.ishaqparveen@meduniwien.ac.at (I.I.-P.); wolfgang.r.sperr@meduniwien.ac.at (W.R.S.); peter.valent@meduniwien.ac.at (P.V.); 2 Ludwig Boltzmann Institute for Hematology and Oncology, Medical University of Vienna, 1090 Vienna, Austria; mathias.schneeweiss@meduniwien.ac.at (M.S.-G.); gregor.hoermann@mll.com (G.H.); 3 Department of Medicine III, Division of Hepatology and Gastroenterology, Medical University of Vienna, 1090 Vienna, Austria; 4 Faculty of Pharmaceutical Sciences, Sojo University, Kumamoto 860-0082, Japan; 5 Division of Gastroenterology and Hepatology, Johns Hopkins University School of Medicine, Baltimore, MD 21205, USA; bcmb.waliul@gmail.com; 6 Department of Hematology and Oncology, University Hospital Mannheim, Heidelberg University, 68167 Mannheim, Germany; juliana.schwaab@umm.de (J.S.); andreas.reiter@umm.de (A.R.); 7 Departments of Oncology and Cell Biology, Albert Einstein College of Medicine, Bronx, NY 10461, USA; marina.konopleva@einsteinmed.edu; 8 Department of Leukemia, University of Texas, MD Anderson Cancer Center, Houston, TX 77030, USA; mandreef@mdanderson.org; 9 MLL Munich Leukemia Laboratory, 81377 Munich, Germany; 10 French Reference Network for Mastocytosis (CEREMAST) and Department of Hematological Biology, Pitié-Salpêtrière Hospital, AP-HP, Paris Sorbonne University, 75013 Paris, France; michelarock0@gmail.com; 11 Research Institute of Molecular Pathology (IMP), Vienna BioCenter (VBC), 1030 Vienna, Austria; 12 Medical University of Vienna, Vienna BioCenter (VBC), 1030 Vienna, Austria; 13 Department of Laboratory Medicine, Medical University of Vienna, 1090 Vienna, Austria

**Keywords:** systemic mastocytosis, bardoxolone methyl (CDDO-Me), omaveloxolone, triterpenoids, HO-1, KIT D816V inhibitors, drug synergism, SMA-ZnPP

## Abstract

**Highlights:**

**What are the main findings?**

- Neoplastic mast cells exhibit high sensitivity to triterpenoids.
- Triterpenoids act synergistically with KIT inhibitors to suppress the proliferation of neoplastic mast cells.

**What are the implications of the main findings?**

- The combination of triterpenoids and KIT inhibitors warrants evaluation in clinical trials for advanced systemic mastocytosis.
- Triterpenoids may enhance therapeutic efficacy in the treatment of advanced systemic mastocytosis.

**Abstract:**

Advanced systemic mastocytosis (advSM), including aggressive SM (ASM), SM with an associated hematologic neoplasm (SM-AHN) and mast cell leukemia (MCL), are rare, mostly KIT D816V-driven malignancies with a poor prognosis. KIT D816V-targeting drugs, such as midostaurin or avapritinib, can induce hematologic responses in most patients with advSM. However, relapses may occur. Therefore, current research focuses on novel drugs and drug combinations to improve outcomes in advSM. We here describe that the synthetic triterpenoids bardoxolone methyl and omaveloxolone suppress proliferation in various human MCL-like cell lines expressing or lacking *KIT* D816V, with IC_50_ values of 0.2–0.4 µM. Growth-inhibitory effects were accompanied by induction of apoptosis, decreased surface expression of CD44, CD71 and KIT, and downregulation of cytoplasmic AKT. Antiproliferative effects of bardoxolone methyl and omaveloxolone were also observed in primary neoplastic cells obtained from patients with advSM. Moreover, both synthetic triterpenoids were found to cooperate with midostaurin and avapritinib in suppressing the proliferation of HMC-1 and MCPV-1 cells. However, bardoxolone methyl and omaveloxolone also augmented expression of heme oxygenase-1 (HO-1), a mediator of cell survival and drug resistance in these cells. The HO-1 inhibitor SMA-ZnPP was subsequently applied and found to sensitize neoplastic mast cells against synthetic triterpenoids and KIT D816V-targeting drugs. Together, application of bardoxolone methyl and omaveloxolone, as single agents or in drug combinations involving HO-1 blockers and/or KIT D816V-targeting drugs, may be a suitable strategy to improve therapy and to overcome resistance in advSM. The clinical value of this combination approach remains to be assessed in clinical trials.

## 1. Introduction

Systemic mastocytosis (SM) is a rare hematopoietic neoplasm characterized by the expansion of neoplastic mast cells (MCs) in various internal organs [1,2,3,4,5,6]. In most cases, neoplastic MCs express the D816V-mutated variant of KIT, which is considered a major driving oncoprotein in SM [1,2,3,4,5,6]. In contrast to patients with indolent SM (ISM) or smoldering SM (SSM), who often suffer from mediator symptoms but usually do not develop a leukemic disease and have a near normal life expectancy, patients with advanced SM (advSM), including SM with an associated hematologic neoplasm (SM-AHN), aggressive SM (ASM), and MC leukemia (MCL), have a poor prognosis [1,2,3,4,5,6,7,8,9]. In these patients, life expectancy is compromised by destructive MC proliferation or a leukemic course, and cytoreductive therapies are needed to reduce the MC burden and improve survival. Established treatment strategies for such patients include polychemotherapy, cladribine, other cytoreductive drugs, and allogeneic hematopoietic stem cell transplantation, which can, however, only be offered to younger and fit patients [3,5,10].

In the last decade, KIT D816V-targeting tyrosine kinase inhibitors (TKIs) have been successfully developed for the treatment of advSM. Midostaurin, which was approved in 2017 for the treatment of patients with advSM, can reduce the MC burden and induces clinical responses in a majority of patients with advSM [3,11]. However, hematologic remissions are usually not achieved, and relapse or progression occurs frequently [3,5,7,10]. Avapritinib, a second-generation KIT D816V TKI, has also received approval for treatment of patients with advSM. Clinical studies have shown that avapritinib is more effective in the treatment of advSM compared to midostaurin. Indeed, avapritinib reportedly induces long-lasting hematologic remissions in a subset of patients with advSM [12,13]. In addition, avapritinib is effective in patients with symptomatic ISM [14]. More recently, several additional TKIs targeting KIT D816V or related signaling pathways have been developed and are currently being tested in preclinical and clinical studies [3,15,16,17,18,19,20].

However, relapse or disease progression may occur in all patient subsets, independent of the type of SM, signaling pathways involved in cell expansion, and TKI applied [5,6,7,8,9,10]. Indeed, several different oncogenic signaling pathways may confer resistance to KIT-targeting drugs in patients with advSM [15,16,17,20]. Therefore, current research focuses on the development of novel therapeutic approaches, such as drug combinations, including KIT D816V TKIs and other targeted or conventional drugs.

Synthetic triterpenoids, including bardoxolone methyl and omaveloxolone, are oleanane triterpenoids that have been described to induce ROS generation and to suppress numerous survival-related molecules, including MAPK, AKT, mTOR, NF-κB and STAT3, in various cells [21,22,23,24,25,26,27,28,29,30,31,32,33,34]. Both drugs have been evaluated as immunomodulators in diabetic nephropathy, Alport syndrome, and Friedreich ataxia (FA) [31,32,33,34,35,36,37]. Based on clinical trials, omaveloxolone received approval for treatment in FA [36,37]. Furthermore, bardoxolone methyl and omaveloxolone reportedly promote apoptosis in neoplastic cells in various solid tumors and hematologic malignancies [22,23,24,25,26,27,28,29,30,31]. However, the effects of synthetic triterpenoids have not been evaluated in the context of advSM so far.

The aim of the current project was to evaluate the effects of the synthetic triterpenoids bardoxolone methyl and omaveloxolone, alone and in combination with midostaurin, avapritinib and nintedanib, on neoplastic cells in advSM. Since bardoxolone methyl and omaveloxolone also increase expression of heme oxygenase-1 (HO-1), a mediator of drug resistance and cell survival, we also included the HO-1 inhibitor styrene-maleic acid copolymer-conjugated zinc protoporphyrin (SMA-ZnPP) in our investigations [38,39,40].

## 2. Materials and Methods

### 2.1. Isolation of Primary Neoplastic Cells

Primary neoplastic cells were isolated from bone marrow (BM) samples obtained during routine diagnostic investigations in 11 patients with ISM, 1 with SSM, 12 with ASM, 7 with SM-AHN, and 4 with MCL (Table 1), after written informed consent was provided by the patients. For 4 patients, BM samples were collected at two or more time points during follow-up (Table 1). BM samples were layered over Ficoll (PAN-Biotech, Aidenbach, Germany) to isolate mononuclear cells (MNCs) [24]. In addition, normal BM MNCs from 3 healthy donors, obtained from Lonza Biosciences (Walkersville, MD, USA), were used. The study was approved by the ethics committee (EC) of the Medical University of Vienna (EC approval number: #2396/2024; biobank EC approval number: #1184/2014).

### 2.2. Culture of Cell Lines

The human MCL-like cell lines HMC-1.1, HMC-1.2, ROSA^KIT WT^, ROSA^KIT D816V^ and MCPV-1 [41] were used in this study. A detailed characterization of these cell lines and the culture conditions applied are provided in the Supplementary Materials.

### 2.3. Reagents

A description of reagents used in this study is provided in the Supplementary Methods. An overview of the culture reagents and drugs is shown in the Supplementary Table S1.

### 2.4. Evaluation of Drug Effects on Cell Proliferation

Primary neoplastic cells, normal BM MNCs and cell lines were incubated in control medium or various concentrations of bardoxolone methyl or omaveloxolone (0.1–1.5 µM) at 37 °C for 48 hours (h). Thereafter, ^3^H-thymidine uptake was measured, as previously described [24]. Furthermore, HMC-1 and MCPV-1 cells were exposed to various concentrations of bardoxolone methyl or omaveloxolone and KIT inhibitors, either as single agents or in combination at a fixed ratio of drug concentrations, before ^3^H-thymidine uptake was measured. IC_50_ values in cell lines were determined by nonlinear regression (four-parameter sigmoidal model; GraphPad Prism 10). In select experiments, the HO-1 blockers SMA-ZnPP and OB-24 were applied in combination with bardoxolone methyl or omaveloxolone. All experiments were performed in triplicate.

### 2.5. Evaluation of Apoptosis in Drug-Exposed Cells

For flow cytometric determination of apoptosis, cell lines were cultured in control medium or various concentrations of bardoxolone methyl or omaveloxolone (0.05–1 μM) at 37 °C for 72 h. Then, combined Annexin V/DAPI staining was performed before apoptosis was measured by flow cytometry, as described in [24]. Furthermore, cell viability was quantified in the MC gate (CD45+, CD117 high, CD34-), the stem cell gate (SC; CD45 dim, CD34+, CD38-), and the progenitor cell gate (PC; CD45 dim, CD34+, CD38+) in BM samples obtained from a patient with ISM and a patient with SSM. To evaluate the effects of bardoxolone methyl and omaveloxolone in normal cells, BM MNCs of 3 healthy donors were analyzed.

### 2.6. Measurement of Histamine Release

Dextran-enriched blood basophils obtained from 3 patients with ISM and one patient with SM-AHN (Table 1) were incubated in control medium or in the presence of bardoxolone methyl or omaveloxolone (1–10 μM) at 37 °C for 30 min. Then, histamine release was determined, as previously described in [20]. A detailed description of the technique is provided in the Supplementary Materials.

### 2.7. Evaluation of Expression of Activation-Linked Surface Antigens by Flow Cytometry

HMC-1.1 and HMC-1.2 cells were kept in control medium or in the presence of bardoxolone methyl or omaveloxolone (0.5–1 μM) at 37 °C for 24 h. Thereafter, expression of cell surface antigens was analyzed using monoclonal antibodies against CD25, CD44, CD63, CD71, CD95, CD117, and CD203c (Supplementary Table S2).

### 2.8. Western Blot Analysis and Quantitative PCR (qPCR)

To determine drug effects on the expression of KIT, HO-1, and critical signaling molecules, HMC-1 and MCPV-1 cells were kept in control medium or in the presence of bardoxolone methyl or omaveloxolone (0.1–1 µM) for 1–24 h. Then, Western blotting (WB) and qPCR were performed essentially as described previously [24,42]. Western blotting was performed with specific antibodies to quantify the expression of total AKT, phosphorylated (p)-AKT, total KIT, p-KIT, total HO-1 or actin/β-tubulin (loading control) (Supplementary Table S3). qPCR was performed using primers specific for *KIT*, *AKT*, and *HO-1* (Supplementary Table S4). Details are provided in the Supplementary Methods section.

### 2.9. Knockdown of HO-1 with shRNA

HO-1 (*HMOX1*) was silenced using miR-E-based shRNA constructs delivered to HMC-1.2 cells by lentiviral transduction. Viral gene transduction was performed essentially as described in [41,43]. An shRNA targeting *Renilla* luciferase served as the non-targeting control vector [43]. Knockdown efficiency was confirmed by qPCR. Technical details are described in the Supplementary Methods section. Guide sequences are shown in Supplementary Table S5.

### 2.10. Statistical Analysis

To determine the level of significance, the two-tailed Student’s *t*-test or one-way ANOVA with Bonferroni’s correction for multiple comparisons was applied using GraphPad Prism version 10 (GraphPad Software, San Diego, CA, US). Results were considered significantly different when *p* < 0.05. In drug combination experiments, drug interaction types were determined by calculating combination index (CI) values using Calcusyn 2.11 software [44]. A CI of <1 indicates a synergistic drug combination effect. In addition, the type of drug combination effects was analyzed by calculating synergy scores (zero interaction potency, ZIP, and highest single agent, HSA) using SynergyFinder Plus 3.0 software [45,46]. A synergy score > 10 indicates synergism, a score between -10 and 10 an additive effect, and a synergy score < -10 stands for antagonism [45,46].

## 3. Results

### 3.1. Bardoxolone Methyl and Omaveloxolone Inhibit Proliferation and Viability of MCL-like Cell Lines and Primary Neoplastic MCs

**Figure 1:**
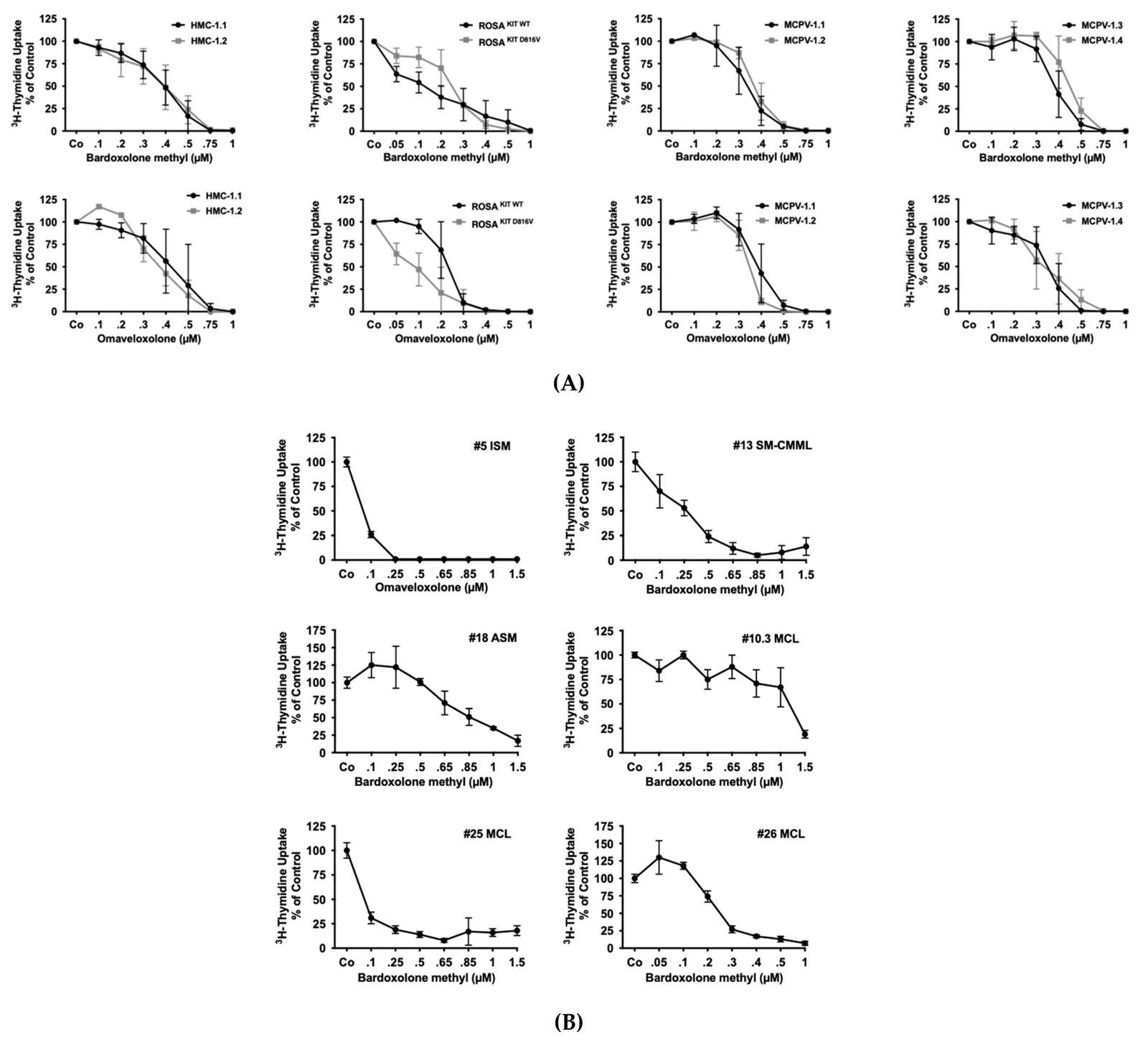
Bardoxolone methyl and omaveloxolone inhibit the proliferation of MCL-like cell lines and primary neoplastic MCs. (**A**) The MCL-related cell lines HMC-1.1, MCPV-1.1, ROSA^KIT WT^ (all *KIT* D816V negative), HMC-1.2 and ROSA^KIT D816V^ (both *KIT* D816V positive) were incubated in control medium (Co) or various concentrations of bardoxolone methyl or omaveloxolone as indicated at 37 °C for 48 h (h). Then, proliferation was measured by assessing ^3^H-thymidine incorporation. Results are expressed in percent of control and represent the mean ± S.D. of 3 independent experiments, each performed in triplicate. (**B**) Primary cells isolated from patients with various types of SM were incubated in control medium (Co) or various concentrations of bardoxolone methyl or omaveloxolone as indicated at 37 °C for 48 h. Then, proliferation was measured by assessing ^3^H-thymidine uptake. Results are expressed in percent of control and represent the mean ± S.D. of triplicates. Patient numbers refer to Table 1.

**Figure 2:**
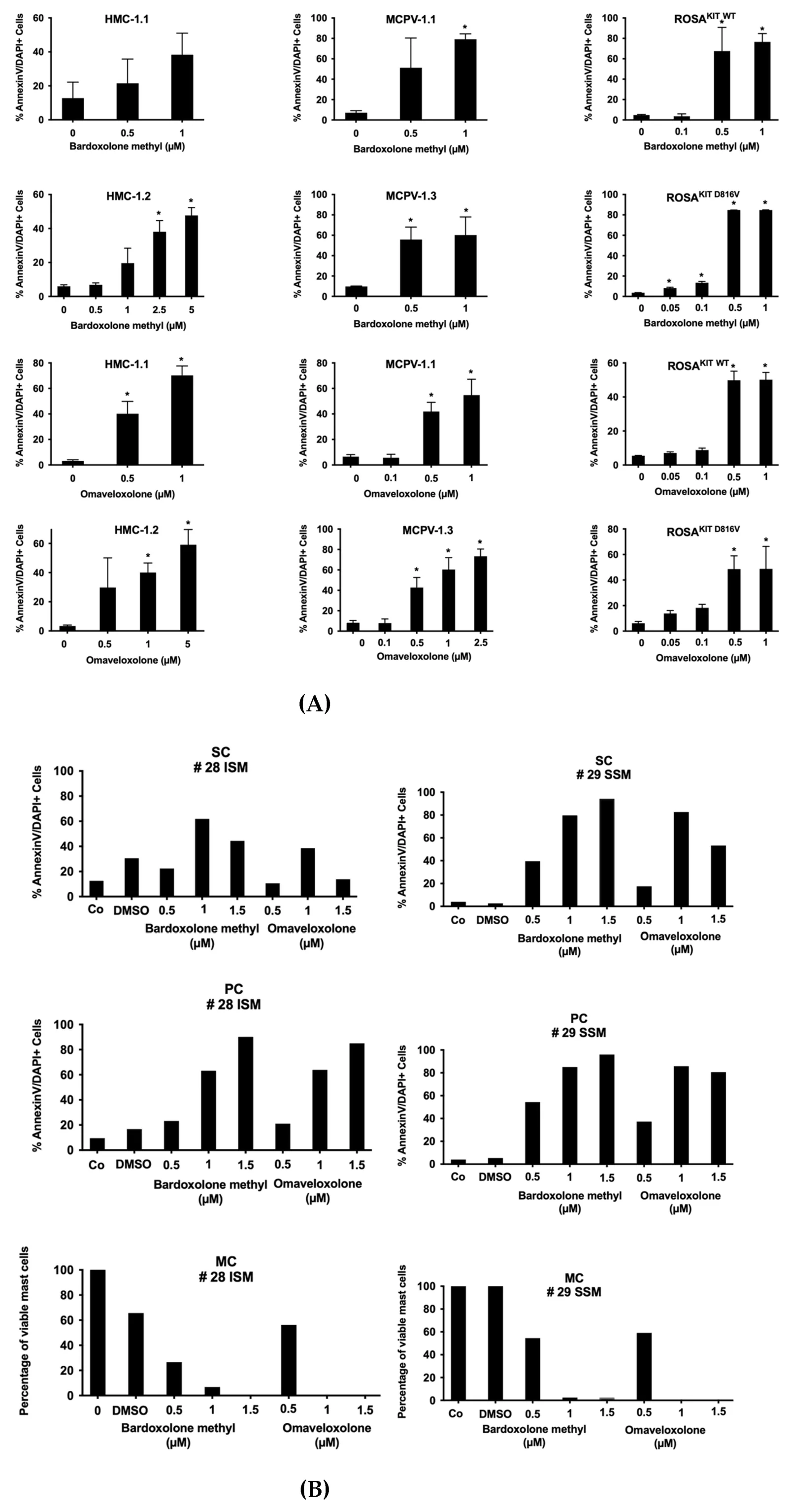

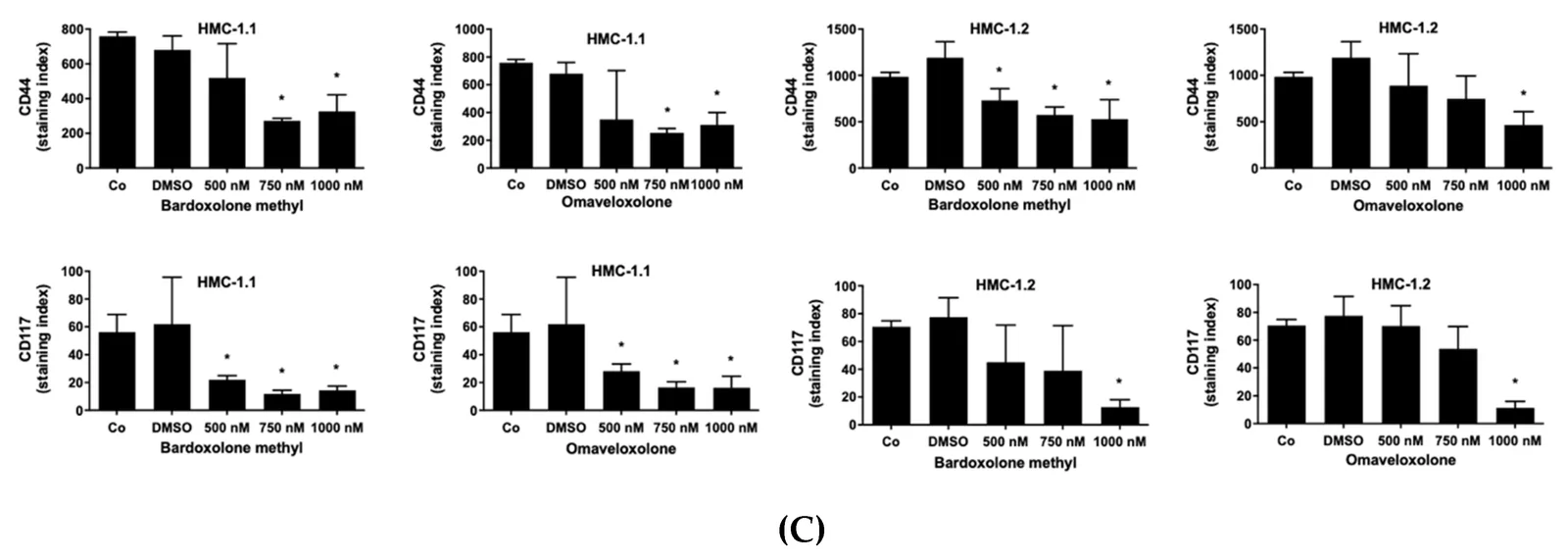
Growth-inhibitory effects of bardoxolone methyl and omaveloxolone in neoplastic mast cells (MCs) are accompanied by induction of apoptosis and inhibition of relevant cell surface antigens in MCL-like cell lines. (**A**) MC lines were incubated in control medium (0) or medium containing various concentrations of bardoxolone methyl or omaveloxolone as indicated at 37 °C for 72 h (h). Thereafter, cells were harvested, and Annexin V/DAPI-positive cells were analyzed by flow cytometry, as described in the Materials and Methods section. Bar graphs show the percentage of apoptotic cells and represent the mean ± S.D. of 3 independent experiments. Statistical significance was determined by one-way ANOVA with Bonferroni’s correction for multiple comparisons; * *p* < 0.05 compared to the control. (**B**) Effects of bardoxolone methyl and omaveloxolone on apoptosis and viability of hematopoietic stem cells (SCs), progenitor cells (PCs), and MCs in patients with systemic mastocytosis (SM). For analysis of apoptosis and viability, bone marrow (BM) cells from one patient with indolent SM (ISM) (left panels) and one with smoldering SM (SSM; right panels) were incubated in control medium (Co), DMSO, or medium containing bardoxolone methyl or omaveloxolone (0.5, 1, or 1.5 µM) as indicated at 37 °C for 48 h. Then, the percentage of apoptotic (Annexin V/DAPI+) cells among all SCs (CD34+/CD38-) and PCs (CD34+/CD38+) was determined by flow cytometry. The percentage of viable MCs (CD117+, CD34-), shown as percentage of Annexin V/DAPI- cells within the MC gate (compared to control) in both patients, is shown in the lower panels. (**C**) HMC-1.1 and HMC-1.2 cells were incubated in control medium, DMSO control medium or medium containing various concentrations of bardoxolone methyl or omaveloxolone as indicated at 37 °C for 24 h. Then, surface expression of CD44 and CD117 was determined by flow cytometry using monoclonal antibodies (mAbs) against indicated antigens. Staining index was defined as the median fluorescence intensity (MFI) of the target mAb divided by the MFI of the isotype-matched control mAb. Results represent the mean ± S.D. of 3 experiments. Statistical significance was determined by two-tailed Student’s *t*-test; * *p* < 0.05 compared to control.

First, we evaluated antiproliferative effects of bardoxolone methyl and omaveloxolone on MCL-like cell lines and primary cells. In ^3^H-thymidine uptake experiments, bardoxolone methyl and omaveloxolone were found to suppress the proliferation of HMC-1.1 (IC_50_: 0.38/0.43 µM), HMC-1.2 (IC_50_: 0.37/0.34 µM), ROSA^KIT WT^ (IC_50_: 0.36/0.32 µM), ROSA^KIT D816V^ (IC_50_: 0.37/0.19 µM), MCPV-1.1 (IC_50_: 0.32/0.38 µM), MCPV-1.2 (IC_50_: 0.37/0.34 µM), MCPV-1.3 (IC_50_: 0.38/0.36 µM) and MCPV-1.4 (IC_50_: 0.44/0.33 µM), with IC_50_ values ranging between 0.2 and 0.4 µM (Figure 1A). No differences were seen between cells lacking or expressing *KIT* D816V or *HRAS* G12V. Next, we examined growth-inhibitory effects of bardoxolone methyl and omaveloxolone in primary neoplastic MCs. In ^3^H-thymidine uptake experiments, we found that bardoxolone methyl blocks the proliferation of neoplastic MCs in a majority of the patient samples tested, with IC_50_ values of 0.1–1 µM (Figure 1B). Omaveloxolone also exhibited strong antiproliferative effects in all tested samples, with IC_50_ values of 0.1–0.25 µM (Figure 1B, Supplementary Figure S1A, Table 1). Interestingly, no differences were observed between subtypes of SM, the presence and type of an AHN, or between MCs isolated from therapy-naïve patients or patients previously treated with midostaurin and/or avapritinib (Figure 1B, Supplementary Figure S1A, Table 1). These data suggest that bardoxolone methyl and omaveloxolone suppress neoplastic MC proliferation in a broad spectrum of patients with advSM. To define mechanisms underlying the growth-inhibitory triterpenoid effects, we explored apoptosis in MCL-like cell lines. As assessed by Annexin V/DAPI staining, both drugs induced apoptosis in a dose-dependent manner in HMC-1.1, HMC-1.2, ROSA^KIT WT^, ROSA^KIT D816V^, and MCPV-1 cells (Figure 2A). We were also able to show that bardoxolone methyl and omaveloxolone induce apoptosis in the stem cell and progenitor cell compartment and even eradicated MCs in one patient with ISM and one with SSM (Figure 2B). By contrast, less potent effects were seen when applying bardoxolone methyl and omaveloxolone on BM MNCs obtained from healthy donors (Supplementary Figure S1B,C).

### 3.2. Bardoxolone Methyl and Omaveloxolone Suppress the Expression of Functionally Relevant Cell Surface Proteins in MCL-like Cell Lines

In advSM, MCs express various surface antigens relevant to MC activation, proliferation and disease expansion [20,47,48,49]. We examined the effects of bardoxolone methyl and omaveloxolone on the expression of CD25, CD44, CD63, CD71, CD95, CD117 and CD203c in HMC-1 cells. In these studies, we found that bardoxolone methyl and omaveloxolone (0.5–1 µM) suppressed the expression of the Hermes invasion receptor CD44, the activation-linked surface antigen CD63, the proliferation-associated transferrin receptor CD71, and KIT (CD117), whereas little to no effects of these drugs on the expression of CD203c, CD25 and CD95 on HMC-1 cells were seen (Figure 2C, Supplementary Figure S2).

### 3.3. Bardoxolone Methyl and Omaveloxolone Inhibit Expression of Total and Phosphorylated KIT and AKT in MCL-like Cell Lines

**Figure 3:**
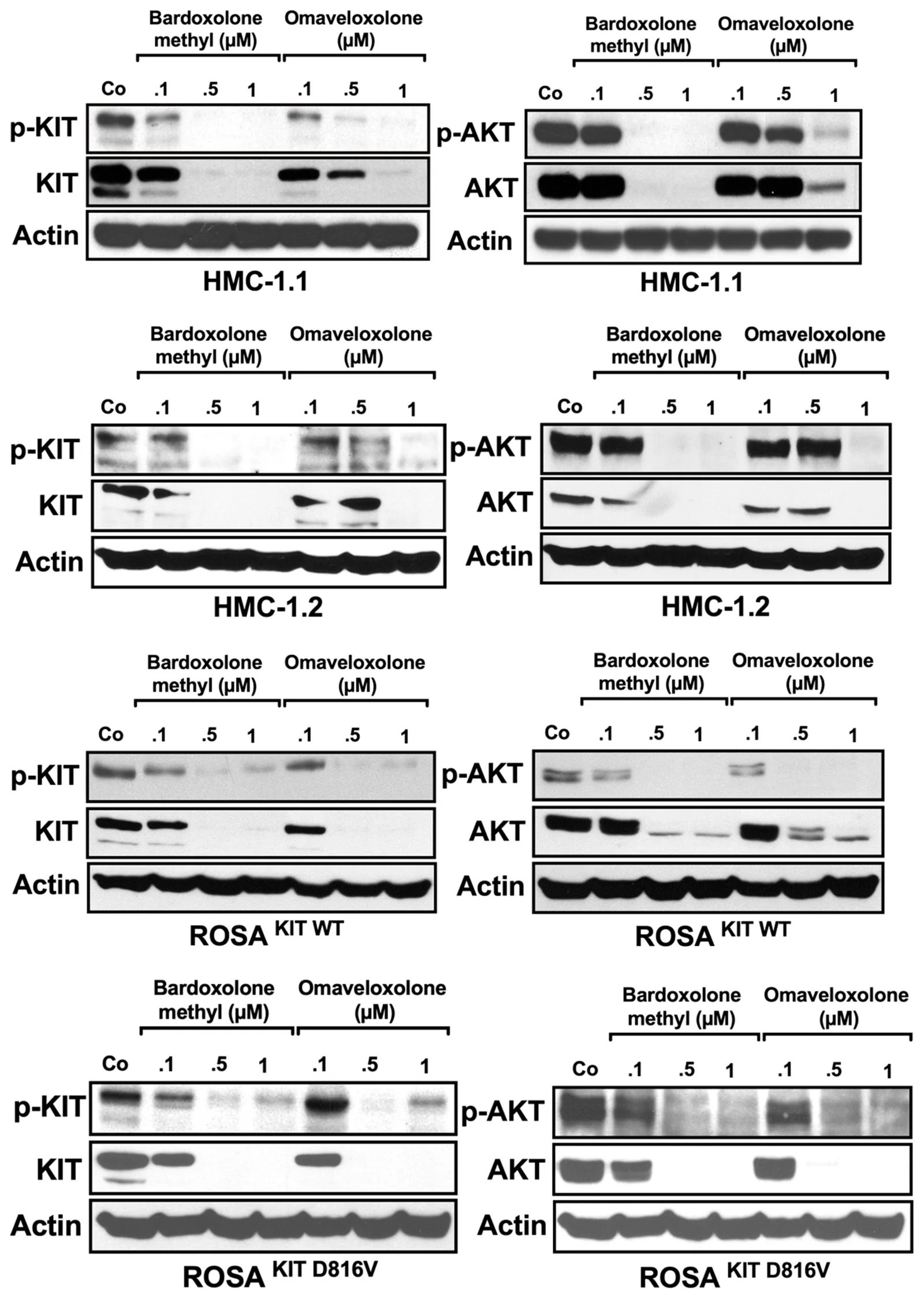
Expression of (p-)KIT and (p-)AKT in neoplastic mast cells is downregulated by treatment with bardoxolone methyl and omaveloxolone. The MCL-related cell lines HMC-1.1, ROSA^KIT WT^ (all *KIT* D816V negative), HMC-1.2 and ROSA^KIT D816V^ (both *KIT* D816V positive) cells were incubated with control medium (Co) or medium containing bardoxolone methyl or omaveloxolone (0.1–1 µM) as indicated at 37 °C for 24 h. Thereafter, cells were harvested, and Western blotting was performed using antibodies against AKT, p-AKT, KIT, p-KIT and actin.

Next, we explored the expression and phosphorylation of pro-oncogenic molecules, including KIT and AKT by WB, in HMC-1.1, HMC-1.2, ROSA^KIT WT^ and ROSA^KIT D816V^ cells exposed to bardoxolone methyl or omaveloxolone. Whereas triterpenoids showed no effects on mRNA levels (Supplementary Materials Figure S3A), we found that both drugs dose-dependently inhibit protein expression of KIT, p-KIT and its downstream targets AKT/p-AKT in all tested MC lines after 24 h (Figure 3). These drug effects were also time-dependent: in fact, after short-term incubation, p-KIT, p-AKT and KIT were downregulated, whereas downregulation of AKT was only observed after 24 h (Supplementary Figure S3B). A densitometric quantification of our Western blot results is shown in Supplementary Figure S3C–G. At all time points (1–24 h), cell counts and cell viability remained stable (Supplementary Figure S3B). Together, these data indicate that bardoxolone methyl and omaveloxolone may not only decrease phosphorylation but also protein expression of KIT, whereas the KIT inhibitors midostaurin and avapritinib primarily inhibit phosphorylation of KIT D816V [20,50,51]. The presence of *KIT* D816V did not affect the suppressive effects of bardoxolone methyl or omaveloxolone on the expression or phosphorylation of KIT and AKT, indicating that mutations in *KIT* do not lead to resistance against synthetic oleanane triterpenoids (Figure 3). Based on the previously described target spectrum of bardoxolone methyl and omaveloxolone, we also analyzed the effects of the drugs on the expression of total and phosphorylated STAT3 and STAT5 in HMC-1 cells. However, no effects of these drugs on STAT3 or STAT5 expression or phosphorylation were found.

### 3.4. Bardoxolone Methyl, but Not Omaveloxolone, Inhibits Histamine Release in Human Basophils Isolated from SM Patients

**Figure 4:**
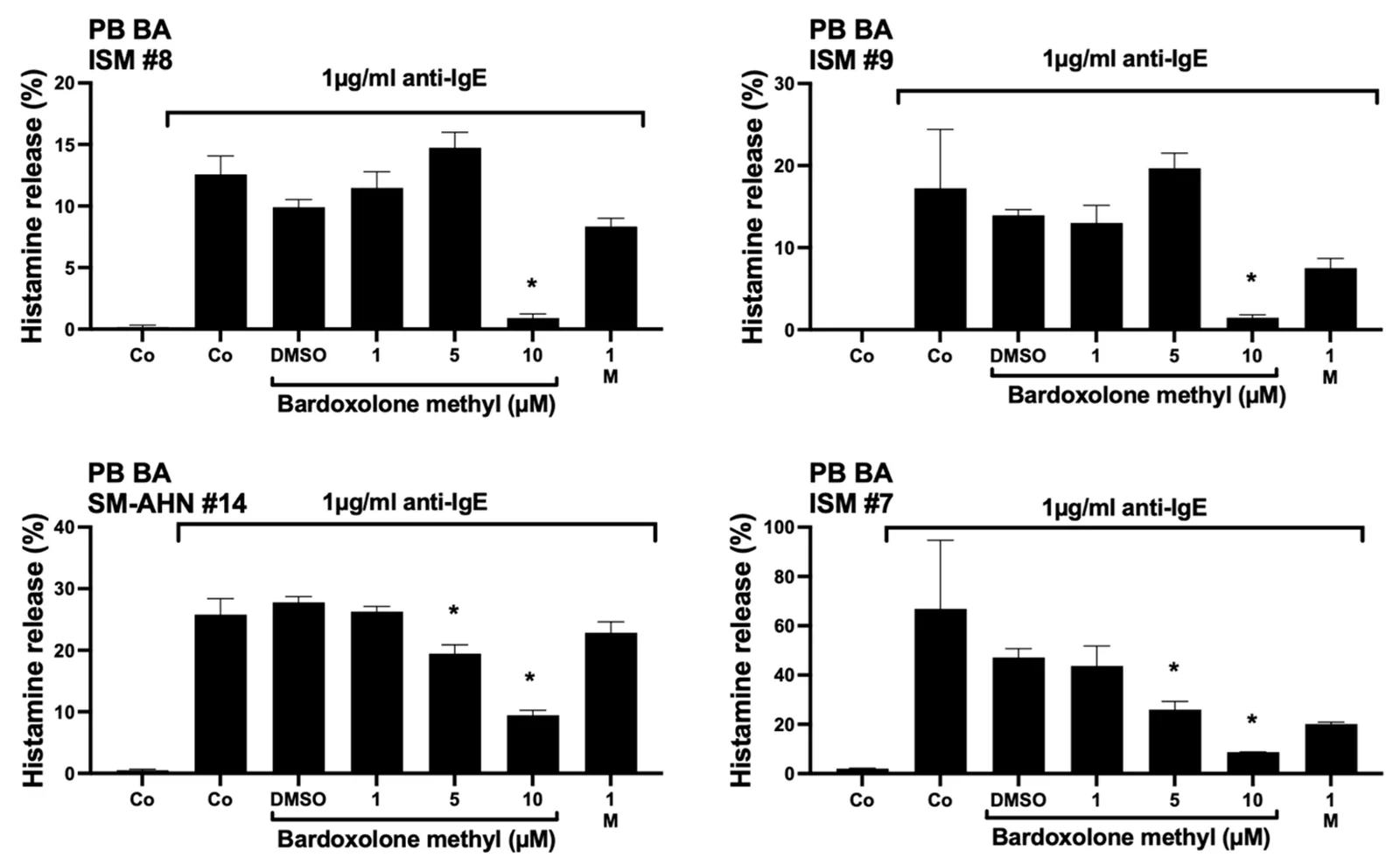
Bardoxolone methyl inhibits histamine release in human basophils isolated from SM patients. Dextran-enriched peripheral blood basophils from SM patients (ISM, *n* = 3; SM-AHN, *n* = 1) were incubated in control medium with or without DMSO or in the presence of bardoxolone methyl (1–10 μM) at 37 °C for 30 min. Then, histamine release was determined by radioimmunoassay. Histamine release was calculated as percent of total histamine and is expressed as percent of control. Results represent the mean ± S.D. of triplicates from single experiments. A total of 1 µM of midostaurin (M) was applied as a positive control. Statistical significance was determined by two-tailed Student’s *t*-test; * *p* < 0.05 compared to control. The patient numbers refer to Table 1.

Patients with SM often suffer from mediator-related symptoms, caused by IgE-dependent or independent activation, degranulation and secretion of mediators [1,2,3,4,5,6,7]. Therefore, we examined the effects of bardoxolone methyl and omaveloxolone on anti-IgE-induced histamine release in human peripheral blood (PB) basophils. Bardoxolone methyl was found to inhibit anti-IgE-induced histamine release in PB basophils obtained from a subset of patients when applied at concentrations above 1 µM (Figure 4). By contrast, omaveloxolone did not inhibit IgE-induced histamine release when applied at therapeutically relevant concentrations. Therefore, it remains uncertain whether bardoxolone methyl can exert any beneficial effects on mediator release and subsequent symptoms in patients with SM.

### 3.5. Bardoxolone Methyl and Omaveloxolone Induce Overexpression of HO-1 and Synergize with the HO-1 Inhibitor SMA-ZnPP in Blocking Cell Proliferation

**Figure 5:**
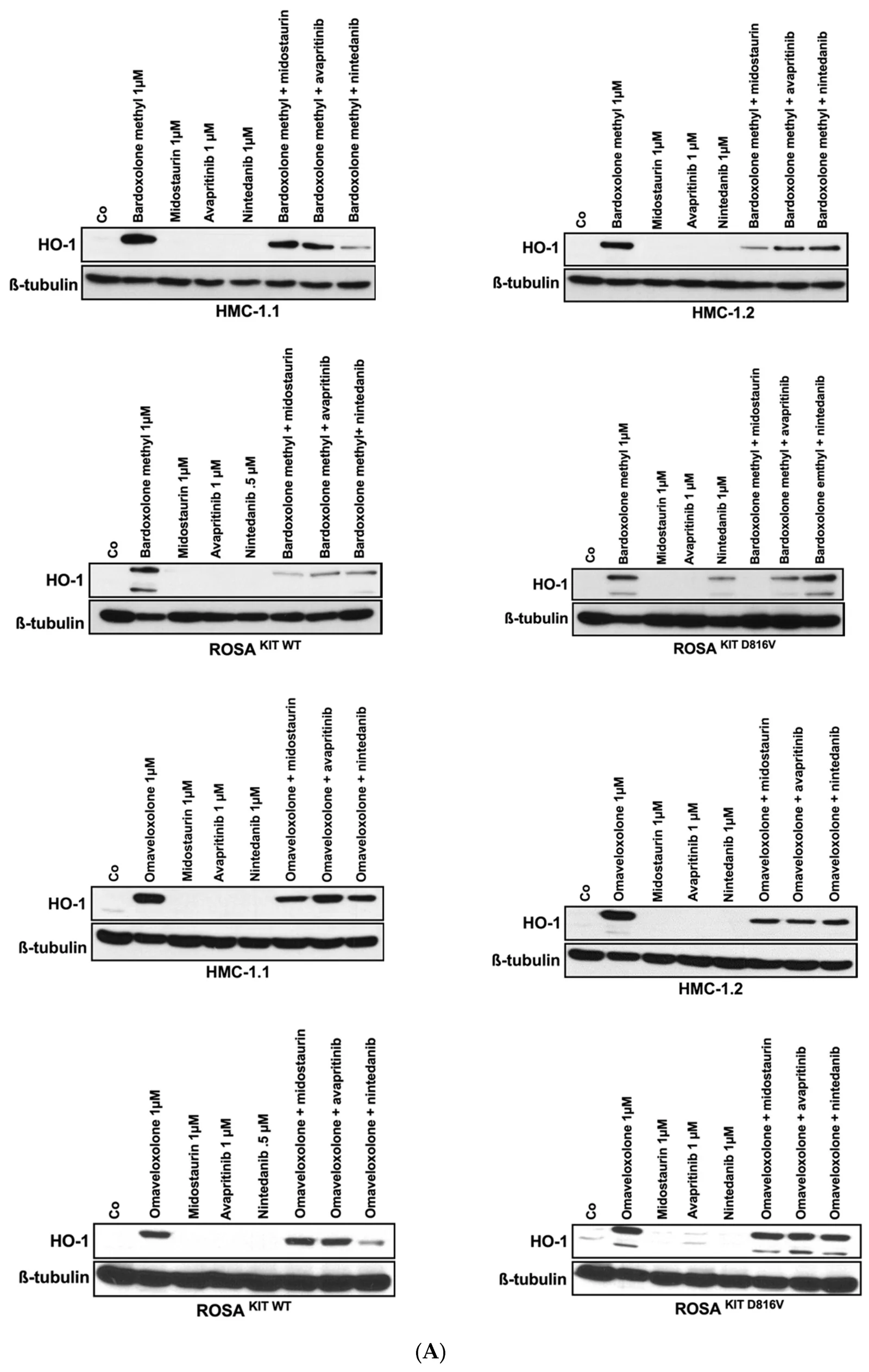

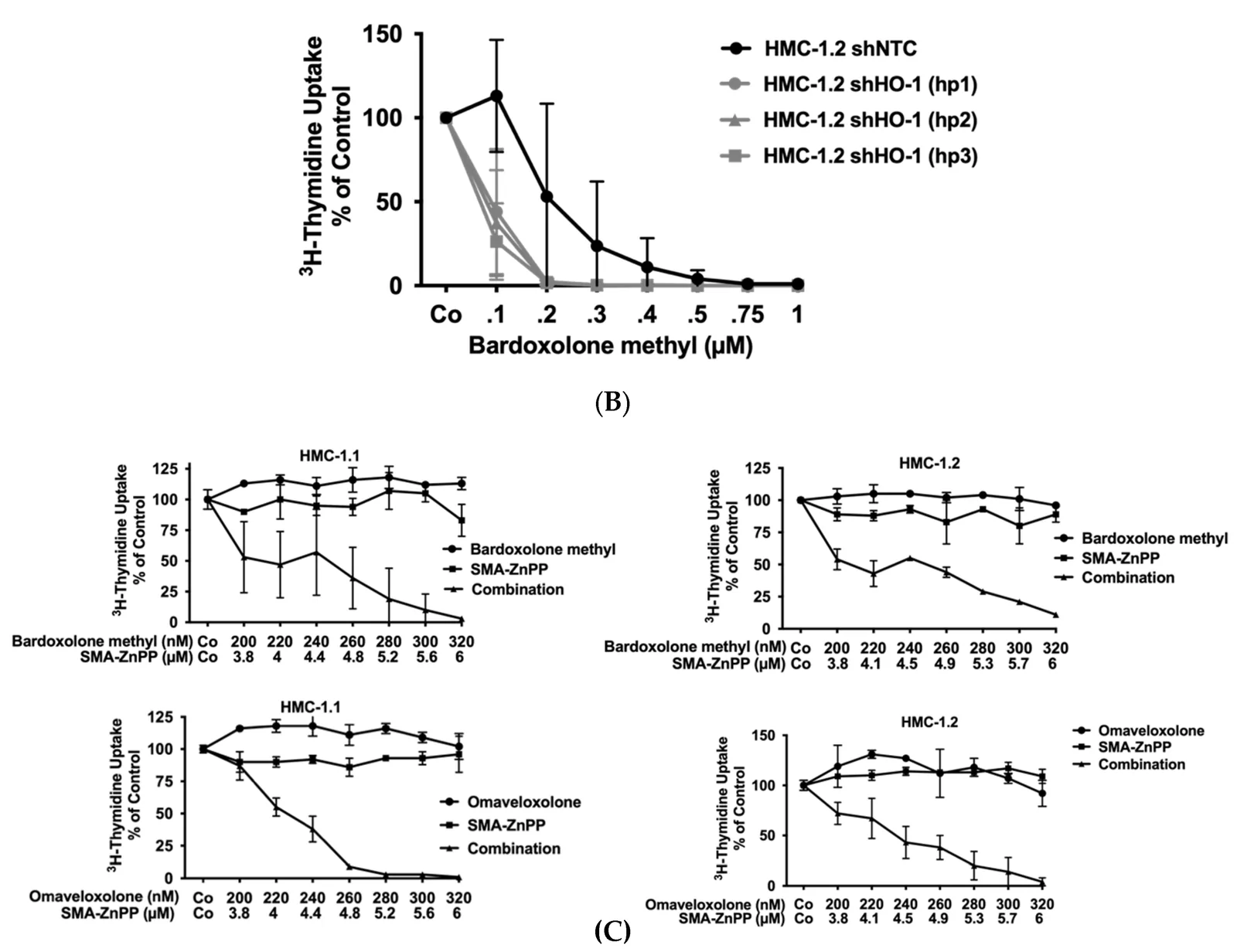
Impact of triterpenoids and KIT-targeting drugs on HO-1 expression and effects of the HO-1 inhibitor SMA-ZnPP in neoplastic MC. (**A**) HMC-1 and ROSA cells were incubated with control medium (Co) or medium containing bardoxolone methyl or omaveloxolone (1µM), midostaurin, avapritinib, or nintedanib (0.5–1 µM), or drug combinations at indicated concentrations at 37 °C for 24 h (h). Thereafter, cells were harvested, and Western blotting was performed using antibodies against HO-1 or β-tubulin. (**B**) HMC-1.2 cells expressing a non-targeting control shRNA (NTC) or one of three independent shRNAs targeting HO-1 (heme oxygenase-1; hp1, hp2, hp3) were incubated in control medium (Co) or 0.1–1 µM bardoxolone methyl as indicated at 37 °C for 48 h. Then, proliferation was measured by assessing ^3^H-thymidine incorporation. Results are expressed in percent of control and represent the mean ± S.D. of 3 independent experiments. (**C**) Effects of the HO-1 inhibitor SMA-ZnPP in human MC lines. HMC-1.1 and HMC-1.2 cells were incubated in control medium (Co) or medium containing either bardoxolone methyl/omaveloxolone or the HO-1 inhibitor SMA-ZnPP alone, or a combination of both drugs (at fixed ratio) for 48 h. Then, proliferation was measured by assessing ^3^H-thymidine uptake. Results show the percentage of ^3^H-thymidine uptake compared with medium control and represent the mean ± S.D. of triplicates from 1 typical experiment. At least two additional experiments with corresponding results were performed for all combinations shown.

Although synthetic triterpenoids suppress certain proteins relevant for malignant MC expansion, including KIT and AKT, these drugs may not target other pro-oncogenic molecules, or they may even upregulate their expression. It has recently been shown that bardoxolone methyl enhances the expression of the stress-related pro-survival molecule HO-1 in the context of leukemic cells [24,29]. We therefore performed WB experiments in HMC-1.1, HMC-1.2, ROSA^KIT WT^, ROSA^KIT D816V^ and MCPV-1 cells. In these experiments, we found that bardoxolone methyl and omaveloxolone enhance expression of HO-1 in all MCL-like cell lines, whereas this effect was not observed with TKI (Figure 5A). This tumor escape mechanism may decrease antineoplastic effects of bardoxolone methyl and omaveloxolone. Indeed, knockdown of HO-1 using shRNA sensitized HMC-1.2 cells against treatment with bardoxolone methyl (Figure 5B, Supplementary Figure S4A–D). We next evaluated cooperative effects of bardoxolone methyl/omaveloxolone and the HO-1 inhibitor SMA-ZnPP. Indeed, this combination resulted in strong cooperative antiproliferative effects in all cell lines tested (Figure 5C, Supplementary Figure S5A and Table S6). Similar effects were seen when using OB-24, another well-known inhibitor of HO-1 (Supplementary Figure S5B).

### 3.6. Bardoxolone Methyl and Omaveloxolone Synergize with Midostaurin, Avapritinib, and Nintedanib in Counteracting Proliferation in Neoplastic MC

**Figure 6:**
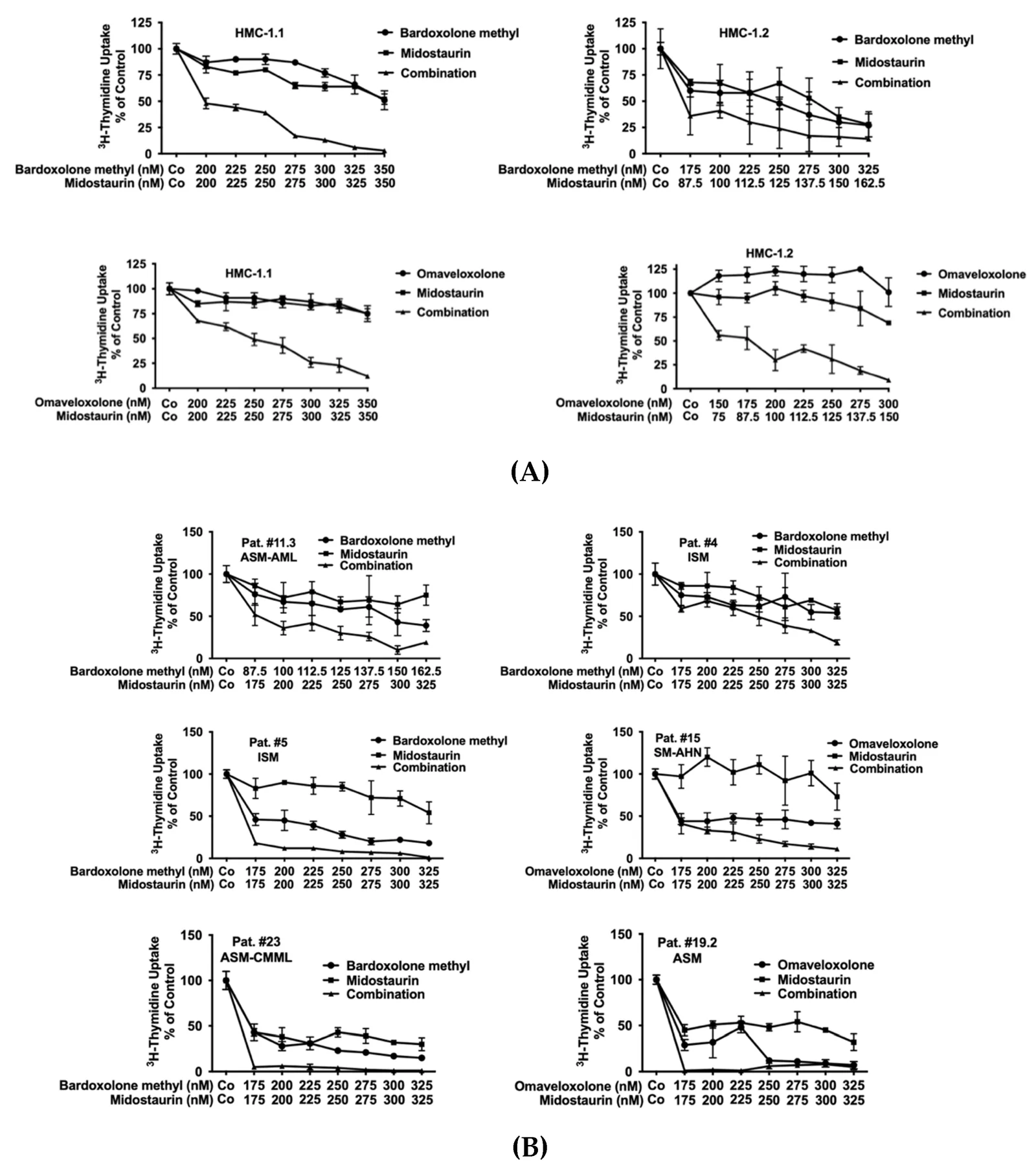

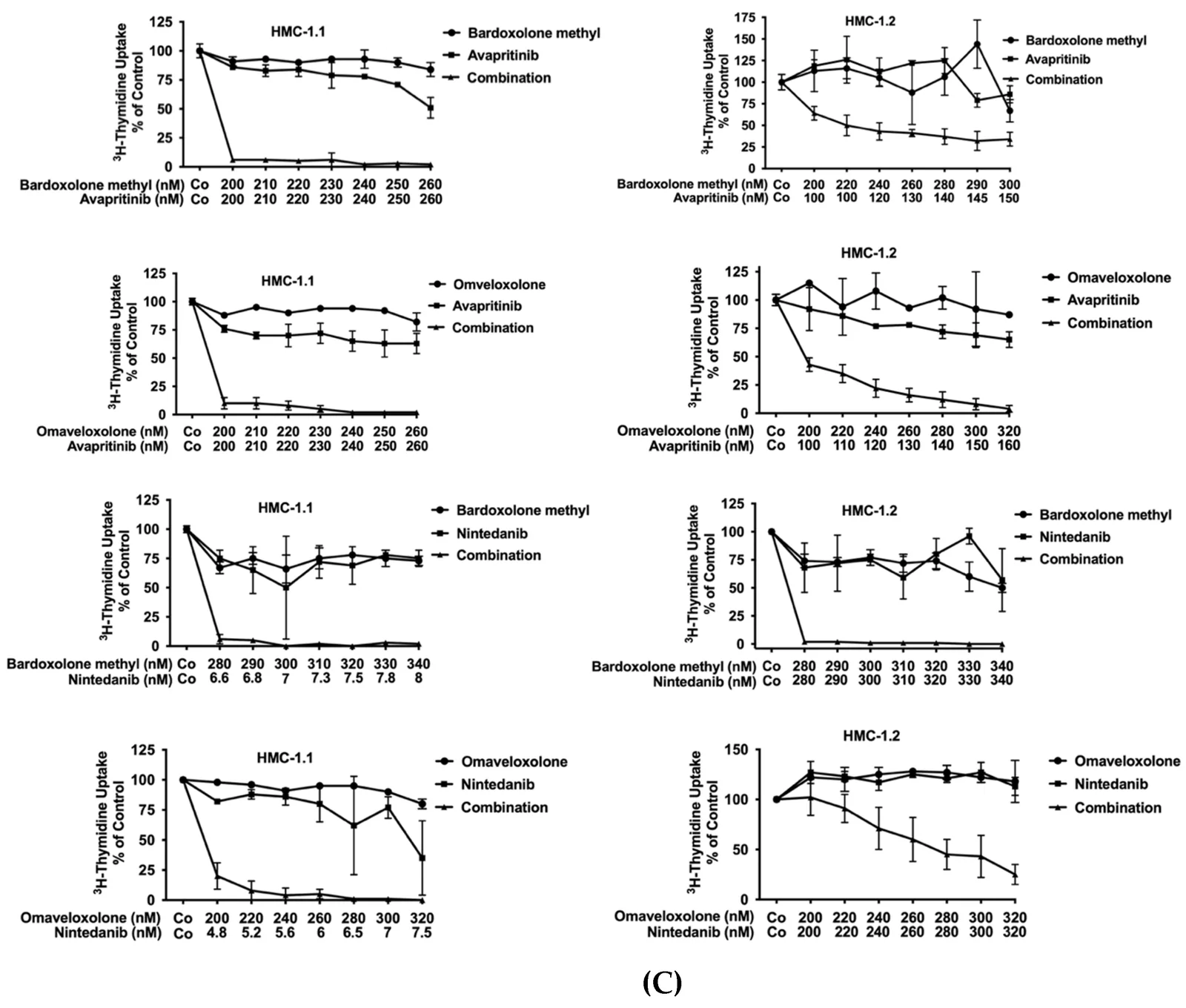
Bardoxolone methyl and omaveloxolone produce cooperative growth-inhibitory effects with midostaurin, avapritinib and nintedanib. (**A**) MC line cells were incubated in control medium (Co) or medium containing either bardoxolone methyl, omaveloxolone or midostaurin alone, or a combination (at fixed ratio) of both drugs (at the concentrations indicated) for 48 h (h). Then, proliferation was measured by assessing ^3^H-thymidine uptake. Results show the percentage of ^3^H-thymidine uptake compared with medium control and represent the mean ± S.D. of triplicates from 1 typical experiment. At least two additional experiments with corresponding results were performed for all combinations shown. (**B**) Primary neoplastic cells from SM patients (ISM: *n* = 2; advSM: *n* = 4; patient numbers refer to Table 1) were incubated in control medium (Co) or medium containing either bardoxolone methyl, omaveloxolone or midostaurin alone, or a combination (at fixed ratio) of both drugs (at the concentrations indicated) for 48 h. Then, proliferation was measured by assessing ^3^H-thymidine uptake. Results show the percentage of ^3^H-thymidine uptake compared with medium control and represent the mean ± S.D. of triplicates. (**C**) MC line cells were incubated in control medium (Co) or medium containing either bardoxolone methyl, omaveloxolone or avapritinib and nintedanib alone, or a combination (at fixed ratio) of both drugs (at the concentrations indicated) for 48 h. Then, proliferation was measured by assessing ^3^H-thymidine uptake. Results show the percentage of ^3^H-thymidine uptake compared with medium control and represent the mean ± S.D. of triplicates from 1 typical experiment. At least two additional experiments with corresponding results were performed for all combinations shown.

Next, we asked whether other drugs, including KIT-targeting TKI, would counteract the HO-1-upregulating and thus the anti-apoptotic effect of synthetic triterpenoids in neoplastic MC and thus would restore their full antineoplastic potential. We found that midostaurin, avapritinib and nintedanib reversed the effects of bardoxolone methyl and omaveloxolone on HO-1 expression (Figure 5A), indicating that midostaurin, avapritinib and nintedanib may be suitable combination partners for bardoxolone methyl and omaveloxolone in neoplastic MC. These encouraging observations prompted us to investigate potential cooperative or even synergistic effects between bardoxolone methyl or omaveloxolone and midostaurin on cell proliferation. In particular, we performed ^3^H-thymidine uptake experiments in MCL-like cell lines exposed to suboptimal concentrations of bardoxolone methyl or omaveloxolone and midostaurin as single agents or in combination. In these experiments, bardoxolone methyl and omaveloxolone produced additive or even synergistic effects with midostaurin in blocking the growth of HMC-1 and MCPV-1 cells (Figure 6A), as well as primary neoplastic cells obtained from patients with ISM or advSM (Figure 6B). Cooperative effects of these drug combinations were confirmed by CalcuSyn 2.11 software (Supplementary Table S6 and Figure S6A–D), as well as by SynergyFinder Plus 3.0 software (Supplementary Table S6). Next, we were interested in potential cooperative effects between bardoxolone methyl/omaveloxolone and the second-generation KIT TKIs avapritinib and nintedanib. Bardoxolone methyl and omaveloxolone synergized with both avapritinib and nintedanib in inhibiting the growth of HMC-1 and MCPV-1 cells (Figure 6C, Supplementary Figure S6E and Table S6). Together, these data suggest that synthetic triterpenoids could serve as suitable combination partners for KIT-targeting TKIs in neoplastic MCs.

### 3.7. SMA-ZnPP Enhances the Growth-Inhibitory Effects of the “Triterpenoid + TKI” Combinations in Neoplastic MC

**Figure 7:**
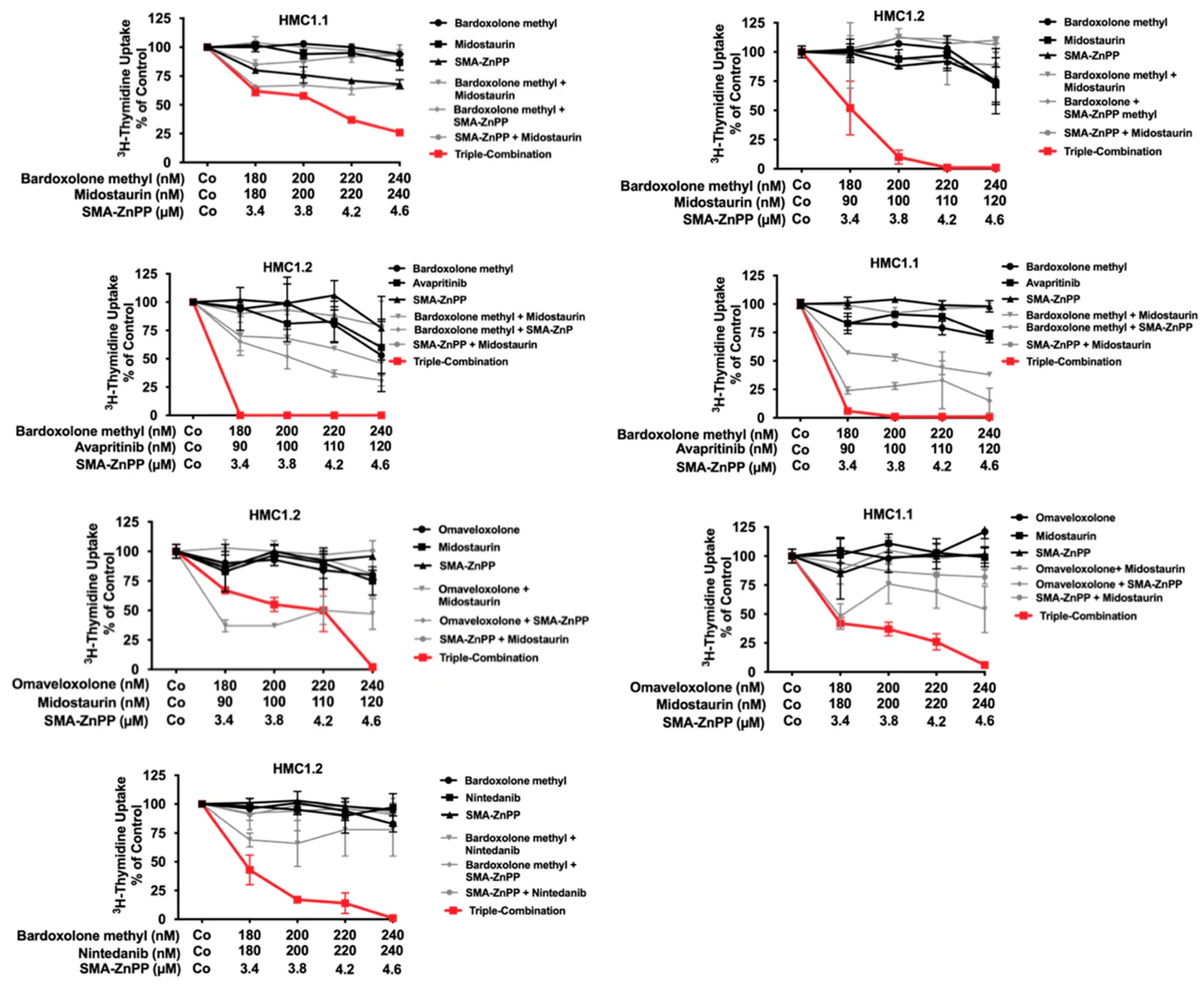
SMA-ZnPP sensitizes neoplastic mast cells against the combination “synthetic triterpenoid + KIT TKI”. HMC-1.1 and HMC-1.2 cells were kept in control medium (Co) or in the presence of a single drug ((black lines: bardoxolone methyl/omaveloxolone (●-●); one of the TKI: midostaurin/avapritinib/nintedanib (■-■); SMA-ZnPP (▲-▲)), a two-drug combination ((grey lines: “Bardoxolone methyl/omaveloxolone + midostaurin/avapritinib/nintedanib” (▼-▼); “Bardoxolone methyl/omaveloxolone + SMA-ZnPP” (⬧-⬧); “SMA-ZnPP + midostaurin/avapritinib/nintedanib” (●-●)) or the three-drug combination “bardoxolone methyl/omaveloxolone + midostaurin/avapritinib/nintedanib + SMA-ZnPP” (red line; □-□) for 48 h before ^3^H-thymidine uptake was evaluated. Results are calculated as percent of control and represent the mean ± S.D. of triplicates of one representative experiment. At least two additional experiments with corresponding results were performed for all combinations shown.

As bardoxolone methyl and omaveloxolone were found to cooperate/synergize with both KIT-targeting TKIs and the HO-1 inhibitor SMA-ZnPP, we evaluated the triple combinations “bardoxolone methyl/omaveloxolone + midostaurin + SMA-ZnPP”, “bardoxolone methyl/omaveloxolone + avapritinib + SMA-ZnPP”, or “bardoxolone methyl + nintedanib + SMA-ZnPP” in neoplastic MCs. As assessed by ^3^H-thymidine uptake, all tested triple combinations completely blocked cell growth (Figure 7 and Supplementary Figure S7). Our results also show that the triple combination “bardoxolone methyl/omaveloxolone + TKI + SMA-ZnPP” exerts superior antineoplastic effects on MC lines compared to dual-drug combinations.

## 4. Discussion

Synthetic triterpenoids have been investigated in preclinical and clinical studies in various disease models, including solid tumors and non-neoplastic conditions [21,22,23,24,25,26,27,28,29,30,31,32,33,34,35,36]. These agents have also shown antiproliferative effects in various hematologic neoplasms [23,24,30]. However, synthetic triterpenoids have not been evaluated in the context of SM so far. In this study, we show that bardoxolone methyl and omaveloxolone block the proliferation of MCL-like cell lines and of primary neoplastic MCs obtained from patients with SM at pharmacological concentrations. Both drugs were effective regardless of the presence or absence of *KIT* D816V or other rare *KIT* mutations, the presence of oncogenic mutations in other driver genes, the presence and type of an AHN, or resistance to KIT TKIs. In addition, both agents exerted strong growth-inhibitory effects on neoplastic MCs in samples obtained from relapsed or refractory patients with advSM, including MCL, and both agents were found to even suppress growth in the highly resistant *HRAS* G12V-transformed human MCL-like cell line MCPV-1 [41]. These findings suggest that triterpenoids may represent new effective drugs potentially useful for the treatment of advSM, including drug-resistant patients. It is also worth noting that IC_50_ values ranged between 0.1 and 1.0 µM in most cell line models and primary neoplastic cells, which corresponds to drug concentrations that can be reached in vivo in patients without marked toxicity [30,31,32,33,34,35,36,37].

In patients with advSM, not only MCs but also other cell types and neoplastic stem cells are critical targets of therapy [3,47]. Therefore, it was of considerable importance to demonstrate that the triterpenoids applied are not only able to attack neoplastic MCs and drive these cells into apoptosis but may also be able to eliminate the CD34+/CD38- stem cells and the CD34+/CD38+ progenitor cells in these patients. In our proliferation assay, we were not able to show that the triterpenoids applied specifically or preferentially suppress the growth of MC. Rather, we believe that these drugs also exert considerable antiproliferative effects on other neoplastic cells, which may represent an advantage in patients with SM-AHN, where also AHN cells may be targeted and eliminated by these drugs. In this regard, it is also worth noting that SM-AHN cases represent the majority of all patients with advSM. Concerning healthy BM cells, we have previously shown that bardoxolone methyl inhibits growth and viability in normal cells, including CD34+/CD38- stem cells [24]. However, the concentrations required to induce growth arrest or apoptosis in normal cells were higher than the concentrations required to block the growth of neoplastic cells, opening a therapeutic window for these drugs [24]. In the current study, we have extended these experiments to normal BM cells and to certain drug combinations. In these experiments, we found that midostaurin and triterpenoids, when combined, do not exert additional cooperative effects on cell viability, suggesting that this drug combination may not induce major hematologic toxicity. However, the in vivo toxicity profile of this drug combination has to be determined in clinical studies.

The growth and accumulation of neoplastic MCs are regulated by a number of signaling molecules and pathways, several survival molecules, as well as distinct cell surface antigens, such as CD44, CD71 or CD117 (KIT) [20,47,48,52,53,54]. To investigate the mechanisms of action of the synthetic triterpenoids, we examined their effects on expression of relevant cell surface antigens and of KIT downstream signaling molecules. We found that exposure of our MC lines to bardoxolone methyl or omaveloxolone leads to a decrease in surface expression of the Hermes invasion receptor CD44, the transferrin receptor CD71, and KIT. Drug-induced downregulation of CD44 is of particular interest, as overexpression of this antigen on neoplastic MCs has been shown to be associated with disease aggressiveness and a poor prognosis in patients with SM [47,48]. Furthermore, CD44 may play a role in the homing of neoplastic stem cells and MC expansion, as well as drug resistance [47,48].

KIT activation is a critical step in pro-survival signaling pathways, leading to the expansion of neoplastic MCs [49,50,51]. We asked whether the triterpenoid-induced growth inhibition is associated with a decrease in KIT phosphorylation and KIT downstream signaling in neoplastic MCs. Indeed, both bardoxolone methyl and omaveloxolone were found to suppress expression of phosphorylated KIT (p-KIT) and downstream AKT (p-AKT) in HMC-1 and ROSA cells. However, our data also suggest that bardoxolone methyl and omaveloxolone suppress total KIT and total AKT expression rather than their phosphorylation in neoplastic MCs. These drug effects apparently differ from the effects of KIT D816V-targeting TKIs, which specifically inhibit phosphorylation of KIT and AKT without blocking total KIT and AKT protein expression in neoplastic MCs. These different drug actions may explain why these drug classes, when combined, produced synergistic effects on the proliferation of neoplastic MCs in our experiments. However, apart from KIT and downstream AKT, a number of additional pathways and molecules may contribute to MCs survival and drug resistance in SM, such as the MAP kinase pathway, other signaling molecules, certain transcription factors, and altered cytokine-mediated regulatory pathways in MCs [55,56,57,58].

Previous studies have shown that bardoxolone methyl downregulates STAT3 activation in CML cells [24]. In the current study, we asked whether bardoxolone methyl or omaveloxolone would suppress the expression and phosphorylation of STAT3 and/or STAT5 in neoplastic MCs. However, we found no effects of these drugs on STAT3 or STAT5 expression or activation in neoplastic MCs. These findings indicate that the molecular targets of synthetic triterpenoids vary depending on the disease model analyzed and the cellular background, and that STAT3 and STAT5 are no major targets of bardoxolone methyl or omaveloxolone in neoplastic MCs.

Previous observations in other cell types, including CML cells, demonstrated that exposure to synthetic triterpenoids leads to upregulation of the anti-apoptotic molecule HO-1 [24,29]. Since HO-1 is a well-known mediator of drug resistance, its upregulation induced by synthetic triterpenoids may be part of a tumor escape mechanism [38,39,40]. Indeed, we found that synthetic triterpenoids upregulate HO-1 expression in all MC lines tested. Therefore, we also applied an HO-1-targeting drug with the idea to prevent or overcome drug resistance. In these experiments, the HO-1 blocker SMA-ZnPP was found to synergize with both bardoxolone methyl and omaveloxolone in inhibiting the proliferation of MCL-like cell lines and to sensitize these cell lines against the combination “synthetic triterpenoid + KIT-TKI”. Whether SMA-ZnPP or other HO-1 inhibitors are useful to overcome HO-1-mediated resistance caused by synthetic triterpenoids in SM remains at present unknown. In addition, it remains uncertain which HO-1-targeting drugs can be developed far enough to reach clinical application.

An alternative approach would be to apply drugs that counteract HO-1 expression in neoplastic MCs. Indeed, we found that the KIT D816V-targeting drugs avapritinib and midostaurin to a certain extent counteract synthetic triterpenoid-induced upregulation of HO-1 expression in neoplastic MCs. Based on these observations, we focused on various drug combinations involving triterpenoids and TKIs. In these studies, we found that synthetic triterpenoids synergize with the KIT D816V-targeting drugs midostaurin and avapritinib in inhibiting the proliferation of MCL-like cell lines. These results are clinically relevant, especially as KIT D816V-targeting TKIs represent the standard of care in patients with advSM [3,5,6,7,8,9,10,11]. Moreover, many patients with advSM relapse during TKI therapy and are thus candidates for treatment with novel drug combinations known to overcome TKI resistance. Whether indeed drug combinations consisting of synthetic triterpenoids and TKIs are able to overcome resistance against TKIs in patients with advSM remains unknown. It also remains unknown which TKI may exert the most beneficial effects in such combinations. Midostaurin exhibits a broader spectrum of kinase and non-kinase targets and may therefore be more effective in patients with SM-AHN or other forms of multi-mutated advSM. Moreover, in contrast to avapritinib, midostaurin can be offered to advSM patients with severe thrombocytopenia [3,11,12,13]. Avapritinib, on the other hand, is more effective in suppressing *KIT* D816V+ subclones and may be better tolerated in terms of gastrointestinal side effects compared to midostaurin [12,13]. Therefore, avapritinib may be an optimal partner for drug combinations in most but not all patients with advSM. Nintedanib, currently approved for treatment of idiopathic pulmonary fibrosis, has also been described to suppress the survival of neoplastic MCs in vitro [18,19,20]. However, so far, nintedanib has not been applied in clinical studies to treat patients with advSM. Omaveloxolone has recently been approved for use in FA and would therefore be available for application in clinical trials in patients with advSM. This drug is also known to be well tolerated in most patients and may therefore indeed be considered in cases with relapsed or drug-resistant SM if conventional treatment options are lacking. However, further preclinical studies and clinical trials are required to clarify the clinical efficacy and tolerability, as well as the overall benefit, of these drugs in patients with advSM.

A major clinical challenge in patients suffering from SM is mediator-related symptoms typically seen in those patients in whom SM is accompanied by MCs activation, often in the context of an IgE-dependent allergy [1,2,3,4,5,6,7]. Therefore, we tested the effects of the synthetic triterpenoids on histamine release and on the expression of activation-linked cell surface antigens. In these experiments, we found that bardoxolone methyl at very high concentrations suppressed histamine release in blood basophils obtained from SM patients, whereas omaveloxolone showed no effects. However, only a limited number of cases were tested, and the concentrations of bardoxolone methyl required to suppress histamine release in basophils were quite high. Moreover, only basophils but no MCs were analyzed in this study. On the other hand, bardoxolone methyl and omaveloxolone suppressed the expression of the activation-linked surface antigen CD63 in HMC-1.2 cells. However, experimental results using in vitro models may not fully recapitulate the situation in human patients. Together, it remains unknown whether triterpenoids can block basophil or MC activation in vivo in patients with SM.

Together, bardoxolone methyl and omaveloxolone exhibit antineoplastic effects in neoplastic MCs in SM and synergize with KIT TKIs and with the HO-1 blocker SMA-ZnPP in producing growth inhibition. Whether these drugs and drug combinations can be translated into clinical application remains to be determined in clinical studies.

## Figures and Tables

**Table 1:** Patients’ characteristics and in vitro sensitivity of primary neoplastic cells against bardoxolone methyl and omaveloxolone.

Patient No.	Age	m/f	Diagnosis, SM Variant	*KIT* D816V	Additional Mutated Genes Detected	Serum Tryptase ng/mL	BM MNC % of MC	BM MC Infiltration %	Bardoxolone Methyl IC_50_ (nM)	Omaveloxolone IC_50_ (nM)	Material
#1	60	f	ISM	+	n.d.	25.4	<1%	10%	650–850 nM	n.d.	BM
#2	80	f	ISM	+	n.d.	82.9	<1%	30%	650–850 nM	n.d.	BM
#3	52	f	ISM	+	n.d.	31.5	<1%	5–10%	250–500 nM	n.d.	BM
#4	22	m	ISM	+	n.d.	55.4	<1%	5%	100–250 nM	100–250 nM	BM
#5	61	f	ISM	+	n.d.	126	3%	30%	650–850 nM	<100 nM	BM
#6	56	m	ISM	+	n.d.	85.4	2%	5%	>1500 nM	n.d.	BM
#7	64	f	ISM	+	n.d.	39.8	n.d.	5–10%	n.d.	n.d.	PB
#8	54	m	ISM	+	n.d.	48.5	n.d.	2%	n.d.	n.d.	PB
#9	56	m	ISM	+	n.d.	15.3	n.d.	<5%	n.d.	n.d.	PB
#10.1	68	m	ISM	+	n.d.	83.8	1%	3–5%	650–850 nM	n.d.	BM
#11.1	70	m	SM-CMML *	+	n.d.	374	<1%	20%	250–500 nM	n.d.	BM
#11.2	74	m	SM-CMML *	+	n.d.	245	<1%	15%	<100 nM	<100 nM	BM
#12	48	m	SM-CMML *	+	n.d.	670	n.d.	60%	250–500 nM	n.d.	BM
#13	66	m	SM-CMML *	+	n.d.	175	3%	20%	250–500 nM	n.d.	BM
#14	44	f	SM-ET *	+	*JAK2* V617F	374	n.d.	15%	n.d.	n.d.	PB
#15	51	f	BMM-CML	-	*BCR::ABL1*	10.4	<1%	5%	<100 nM	100–250 nM	BM
#16	62	m	SM-AML *	+	n.d.	26.2	<1%	3–5%	1000–1500 nM	n.d.	BM
#17	71	f	ASM	+	*TET2*, *SH2B3*, *CUX1*, *CBL*	341	n.d.	20–30%	<100 nM	<100 nM	BM
#10.2	69	m	ASM	+	n.d.	69	<1%	3–5%	250–500 nM	n.d.	BM
#18	68	m	ASM	+	n.d.	650	5%	50%	850–1000 nM	n.d.	BM
#19.1	70	m	ASM	+	n.d.	311	1.4%	5–10%	850–1000 nM	n.d.	BM
#19.2	71	m	ASM	+	*SRSF2, TET2*	53.5	<1%	5–10%	<100 nM	<100 nM	BM
#20	78	m	ASM-MPN-eo	+	n.d.	83	3%	5%	100–250 nM	n.d.	BM
#21	75	m	ASM-MDS/MPN-u	+	n.d.	147	n.d.	1%	not reached	n.d.	BM
#22.1	77	m	ASM-MDS/MPN-u	+	*JAK2* V617F	101	n.d.	50%	850–1000 nM	n.d.	BM
#22.2	77	m	ASM-MDS/MPN-u	+	*JAK2* V617F	69.6	n.d.	50%	650–850 nM	n.d.	BM
#11.3	74	m	ASM-AML	+	*ASXL1*, *CBL*, *PTPN11*, *RUNX1*, *SRSF2*, *TET2*	180	<1%	30%	n.d.	n.d.	BM
#23	65	m	ASM-CMML	+	*SRSF2*, *TET2*	10.7	<1%	10%	<100 nM	100–250 nM	BM
#24	63	m	ASM-AML	+	n.d.	16.5	<1%	10%	850–1000 nM	n.d.	BM
#10.3	70	m	MCL	+	n.d.	94.2	8%	80%	1000–1500 nM	n.d.	BM
#25	91	m	MCL	+	n.d.	49.6	<1%	10%	<100 nM	n.d.	BM
#26	58	m	MCL	+	n.d.	250	n.d.	60%	200–300 nM	n.d.	BM
#27	86	m	MCL-MDS	+	n.d.	180	n.d.	30%	>1500 nM	n.d.	BM
#28	33	f	ISM	-	n.d.	33	<1%	1%	n.d.	n.d.	BM
#29	64	m	SSM	+	*DNMT3A*, *TET2*	151	<1%	30%	n.d.	n.d.	BM

Patients’ diagnoses were established according to WHO criteria. BM infiltration with MCs was determined by immunohistochemistry (KIT or tryptase-stained BM sections) and expressed as percent (%) of all BM cells. Bone marrow mononuclear cells (BM MNCs) were analyzed by immunophenotyping, and the % of MCs was determined. The *KIT* D816V mutation was detected by PCR and additional mutations by next-generation sequencing (NGS) or PCR. Score: +, KIT D816V mutation detected; -, no KIT D816V (or additional mutated genes) detected. Abbreviations: m, male; f, female; BM, bone marrow; PB, peripheral blood; SM, systemic mastocytosis; ISM, indolent systemic mastocytosis; ASM, aggressive systemic mastocytosis; SM-AHN, systemic mastocytosis with an associated hematologic neoplasm; SSM, smoldering SM; AML, acute myeloid leukemia; CML, chronic myeloid leukemia; CMML, chronic myelomonocytic leukemia; BMM, bone marrow mastocytosis; MDS, myelodysplastic neoplasm; ET, essential thrombocythemia; MPN, myeloproliferative neoplasm; MDS/MPN-eo, myelodysplastic/myeloproliferative neoplasm with eosinophilia; MDS/MPN-u, myelodysplastic/myeloproliferative neoplasm unclassified; MCL, mast cell leukemia; MC, mast cell; *, in these cases no further sub-classification of the SM type was possible; n.d., not done; nM, nanomolar. Responses of cells to bardoxolone methyl/omaveloxolone were assessed by ^3^H-thymidine uptake. Responsiveness to bardoxolone methyl/omaveloxolone is expressed as IC_50_; in patients #10, #11, #19, and #22, primary neoplastic BM cells were available at different time points for analysis.

## Data Availability

The data used or analyzed during the study are available from the corresponding author on reasonable request.

## Supplementary Materials

The following supporting information can be downloaded at: https://www.mdpi.com/article/10.3390/cells15201898/s1, Table S1: Culture reagents and drugs used in this study; Table S2: Monoclonal antibodies used in flow cytometry experiments; Table S3: Primary antibodies used in Western blot experiments; Table S4: Primers used for qPCR; Table S5: shRNA guide sequences used for lentiviral knockdown of heme oxygenase-1 (HO-1); Table S6: Cooperative (synergistic or additive) effects between triterpenoids and other drugs (KIT D816V targeting TKI/SMA-ZnPP/OB-24) on proliferation of human MC lines as assessed by 3H-thymidine uptake experiments; Figure S1: Effects of triterpenoids in primary neoplastic and normal BM cells; Figure S2: Triterpenoid effects on expression of surface markers in neoplastic MC; Figure S3: Effects of bardoxolone methyl and omaveloxolone on AKT and KIT signaling; Figure S4: shRNA-mediated knockdown of HO-1 in HMC-1.2 cells; Figure S5: HO-1 blockade sensitizes MC against treatment with triterpenoids; Figure S6: Defining combinatorial effects by CalcuSyn software; Figure S7: Triple combinations of SMA-ZnPP and “synthetic triterpenoid + TKI” in neoplastic MC. References [59,60,61,62] are cited in the supplementary materials.
