# Supplementary material for: Triterpenoid Exposure and HO-1 Blockade Sensitize Neoplastic Mast Cells Against Midostaurin and Avapritinib
Source: Cells. 2026 Oct 19;15(20):1898. doi: 10.3390/cells15201898 (PMC13703063; doi:10.3390/cells15201898)
Supplement: Supplementary file 1 [file cells-15-01898-s001.zip › cells-4190688-supplementary.pdf]

Triterpenoid Exposure and HO-1 Blockade Sensitize Neoplastic Mast Cells Against Midostaurin and Avapritinib

Max Vincent John[1,2], Yüksel Filik Schüppler[1,2], Nadine Witzeneder[1], Mathias Schneeweiss-Gleixner[2,3], Barbara Peter[1,2], Daniela Berger[1], Gabriele Stefanzl[1,2], Isma Ishaq-Parveen[1,2], Jun Fang[4], Waliul Islam[5], Juliana Schwaab[6], Andreas Reiter[6], Marina Konopleva[7], Michael Andreeff[8], Gregor Hoermann[2,9], Michel Arock[10], Johannes Zuber[11,12], Klaus Schmetterer[13], Wolfgang R. Sperr[1,2], Peter Valent[1,2] and Karoline V. Gleixner[1,2,*]

1. Department of Internal Medicine I, Division of Hematology and Hemostaseology, Medical University of Vienna, 1090 Vienna, Austria; max.john@meduniwien.ac.at (M.V.J.); yueksel.filik@lbg.ac.at (Y.F.S.); barbara.peter@lbg.ac.at (B.P.); daniela.berger@meduniwien.ac.at (D.B.); gabriele.stefanzl@meduniwien.ac.at (G.S.); is-ma.ishaqparveen@meduniwien.ac.at (I.I.-P.); wolfgang.r.sperr@meduniwien.ac.at (W.R.S.); pe-ter.valent@meduniwien.ac.at (P.V.)

2. Ludwig Boltzmann Institute for Hematology and Oncology, Medical University of Vienna, 1090 Vienna, Austria; mathias.schneeweiss@meduniwien.ac.at (M.S.-G.); gregor.hoermann@mll.com (G.H.)

3. Department of Medicine III, Division of Hepatology and Gastroenterology, Medical University of Vienna, 1090 Vienna, Austria

4. Faculty of Pharmaceutical Sciences, Sojo University, Kumamoto 860-0082, Japan

5. Division of Gastroenterology and Hepatology, Johns Hopkins University School of Medicine, Baltimore, MD 21205, USA; bcmb.waliul@gmail.com

6. Department of Hematology and Oncology, University Hospital Mannheim, Heidelberg University, 68167 Mannheim, Germany; juliana.schwaab@umm.de (J.S.); andreas.reiter@umm.de (A.R.)

7. Departments of Oncology and Cell Biology, Albert Einstein College of Medicine, Bronx, NY 10461, USA; marina.konopleva@einsteinmed.edu

8. Department of Leukemia, University of Texas, MD Anderson Cancer Center, Houston, TX 77030, USA; mandreef@mdanderson.org

9. MLL Munich Leukemia Laboratory, 81377 Munich, Germany

10. French reference network for mastocytosis (CEREMAST) and Department of Hematological Biology, Pit-ié-Salpêtrière Hospital, AP-HP, Paris Sorbonne University, 75013 Paris, France; michelarock0@gmail.com

11. Research Institute of Molecular Pathology (IMP), Vienna BioCenter (VBC), 1030 Vienna, Austria

12. Medical University of Vienna, Vienna BioCenter (VBC), 1030 Vienna, Austria

13. Department of Laboratory Medicine, Medical University of Vienna, 1090 Vienna, Austria

*. Correspondence: karoline.gleixner@meduniwien.ac.at; Tel.: +43-1-40400-82520; Fax: +43-1-40400-40300

This study was supported by the Medical Scientific Fund of the Mayor of the City of Vienna (grants 17015 and 22028). Peter Valent was supported in part by the Austrian Science Fund (FWF) project DOI 10.55776/PAT6394523.

I. Supplementary Methods

Human cell lines and culture conditions

The human mast cell (MC) lines HMC-1.1 containing *KIT* V560G and HMC-1.2 containing *KIT* V560G and *KIT* D816V were kindly provided by Dr. J.H. Butterfield (Mayo Clinic, Rochester, MN) [S1]. HMC-1.1 and HMC-1.2 cells were cultured in IMDM supplemented with 10% FCS, alpha-thioglycerol, and penicillin/streptomycin. In addition, we used the ROSA cell clones $ROSA^{KIT\ WT}$ and $ROSA^{KIT\ D816V}$ as well as 4 MCPV-1 clones, namely MCPV-1.1, MCPV-1.2, MCPV-1.3, and MCPV-1.4 [S2,S3]. $ROSA^{KIT\ WT}$ and $ROSA^{KIT\ D816V}$ cells were established by lentiviral transduction of MC-committed cord blood progenitor cells cultured in stem cell factor (SCF) as reported [S2]. MCPV-1 cells were derived from SCF-cultured cord blood progenitor cells transformed by *h-TERT*, *Large-T* and oncogenic *HRAS* G12V [S3]. ROSA and MCPV-1 cells were maintained in IMDM with 10% heat-inactivated FCS. In the case of $ROSA^{KIT\ WT}$ and MCPV-1, cells were maintained in SCF-containing supernatant (10%) of Chinese hamster ovary cells transfected with murine *scf* (kit ligand=KL). All cell lines were cultured at 37°C in 95% relative humidity (RH) and 5% CO_2 as reported [S2-S6]. Cells were thawed from an original stock every 4-8 weeks.

Reagents

Bardoxolone methyl, omaveloxolone and OB-24 were purchased from Med Chem Express LLC (NJ, US). Avapritinib and nintedanib were purchased from Selleckchem (Houston, TX, US), and midostaurin from LC Laboratories (Woburn, MA, US). Stock solutions of drugs were prepared by dissolving in dimethyl sulfoxide (DMSO) (Merck,

Darmstadt, Germany). Iscove's modified Dulbecco's medium (IMDM) and penicillin and streptomycin were from Lonza (Verviers, Belgium), fetal calf serum (FCS) from Gibco life technologies (Gaithersburg, MD, US), alpha-thioglycerol from Sigma (St. Louis, MO, US), amphotericin from PAN-Biotech (Aidenbach, Germany) and ^{3}H-thymidine from Perkin Elmer (Boston, MA, US). Annexin V/APC was obtained from Biolegend (San Diego, CA, US). Histamine release buffer (HRB) and histamine radioimmunoassay (RIA) kit were purchased from Immunotech (Marseille, France). SMA-ZnPP was kindly provided by Dr. Jun Fang from the Faculty of Pharmaceutical Sciences, Sojo University, Kumamoto, Japan. An overview of additional reagents and antibodies used in this project is provided in Tables S1, S2 and S3.

Isolation of primary cells

Bone marrow (BM) or peripheral blood (PB) cells were obtained during routine diagnostic investigations after written informed consent was provided by patients. These included 11 patients with indolent systemic mastocytosis (ISM), 1 with smoldering SM (SSM), 12 with aggressive SM (ASM), 7 with SM with an associated hematologic neoplasm (SM-AHN), and 4 with MC leukemia (MCL) (see Table 1 in main document). In 4 patients, BM samples were collected at two or more time points during follow-up (see Table 1 in main document). BM or PB samples were layered over Ficoll (PAN-Biotech, Aidenbach, Germany) to isolate mononuclear cells (MNC) as described previously [S5,S6]. Healthy BM cells were purchased from Lonza Biosciences (Walkersville, MD, US). The study was approved by the ethics committee (ETC) of the Medical University of Vienna (EC approval number: #2396/2024; biobank EC approval

number: #1184/2014) and was conducted in accordance with the Declaration of Helsinki.

Evaluation of drug effects on proliferation

Cell lines or primary neoplastic cells were incubated in control medium or various drugs alone or in combination (see below) in 96-well plates (TPP, Trasadingen, Switzerland) at 37°C, 95% RH, and 5% CO_2 for 48 hours (h). Then, antiproliferative effects were determined by measuring ^{3}H-thymidine uptake as reported [S4,S5]. In brief, after incubation with drugs, 0.5 µCi ^{3}H-thymidine was added for 16 h. Cells were then harvested on filter membranes (Packard Bioscience, Meriden, CT, US) and filter-bound radioactivity was counted in a Beta Counter (Microbeta 2, Perkin Elmer, MA, US).

In a separate phase of experiments, HMC-1, ROSA, and MCPV-1 cells were exposed to various concentrations of bardoxolone methyl or omaveloxolone in combination with either midostaurin, avapritinib, or nintedanib before ^{3}H-thymidine uptake was measured. Drug combinations were applied at a fixed ratio of drug concentrations for 48 h essentially as described [S3,S6]. Combinatorial drug effects (additive versus synergistic) were defined by calculating combination index (CI) values using CalcuSyn software (CalcuSyn; Biosoft, Ferguson, MO, US). A CI value of <1 indicates synergism, and CI values of 1 indicate additive drug effects [S6,S8]. Furthermore SynergyFinder Plus was used to confirm synergism or additive effects [S9,S10]. In subsequent experiments, the HO-1 blocker SMA-ZnPP was combined with either bardoxolone methyl or omaveloxolone. In a separate set of experiments, SMA-ZnPP was added to drug combinations consisting of bardoxolone methyl (or omaveloxolone) and KIT

D816V-targeting drugs (midostaurin, avapritinib, nintedanib). All experiments were performed in triplicates.

Evaluation of apoptosis by flow cytometry

Cell lines were incubated in various concentrations of bardoxolone methyl or omaveloxolone (0.05-1 µM) at 37°C for 72 h. After incubation, the percentage of apoptotic cells was quantified by Annexin V/DAPI staining and flow cytometry essentially as described [S5,S6]. In brief, cells were cultured in control medium or various concentrations of bardoxolone methyl or omaveloxolone at 37°C for 72 h. Then, cells were washed and incubated with Annexin V/APC in binding buffer for 15 minutes. Cells were then washed again and DAPI (4′,6-diamidino-2-phenylindole) was added. After washing, the percentage of apoptotic (Annexin V+/DAPI+) cells was determined on a FACS Canto II (Becton Dickinson, San Jose, CA, US) as previously described [S6]. In primary cells obtained from patients with ISM and SSM, the apoptosis-inducing effects of bardoxolone methyl, omaveloxolone, midostaurin, as well as the combination effects of the triterpenoids (either bardoxolone methyl or omaveloxolone) and midostaurin on stem cells (SC), progenitor cells (PC) and MC were evaluated. In addition, we examined the numbers of viable MC after drug exposure.

Flow cytometry analysis of surface protein expression

Flow cytometry experiments were also performed to quantify the expression of cell surface proteins on HMC-1 cells. Prior to flow cytometry staining, cell lines were incubated in control medium, vehicle control (DMSO, dimethyl sulfoxide), or increasing

concentrations of bardoxolone methyl or omaveloxolone (0.5-1 μM) at 37°C for 24 h. Cells were then harvested and incubated in FcR-blocking reagent (Miltenyi Biotec, Bergisch Gladbach, Germany) for 15 minutes. Then, cells were stained with fluorochrome-labeled monoclonal antibodies (mAb) against CD25, CD44, CD63, CD71, CD95, CD117 and CD203c for 15 minutes. A specification of mAb is provided in Table S2. Staining reactions were controlled by isotype-matched control antibodies. Expression of cell surface antigens was quantified by flow cytometry by assessing median fluorescence intensity (MFI) values on a FACS Canto (Becton Dickinson, San José, CA, US). Expression levels were calculated as staining index (= ratio of MFI obtained with mAb/MFI obtained with isotype-control mAb) as reported [S5,S6].

Measurement of histamine release from basophils obtained from SM patients

Dextran-enriched blood basophils obtained from 4 patients with SM were examined for histamine release. To evaluate drug effects on mediator secretion, dextran-enriched basophils (1.5 x 10^6/ml) were incubated in control medium or in medium containing various concentrations of bardoxolone methyl or omaveloxolone (1-10 μM) at 37°C for 30 minutes. Then, cells were incubated in histamine release buffer (HRB) in the absence or presence of anti-IgE antibody (1 μg/mL) at 37°C for another 30 minutes. Cells were then centrifuged at 4°C and cell-free supernatants and total suspensions were examined for histamine content by RIA (Immunotech, Marseille, France) as reported [S11]. Histamine release was expressed as percent of total (cellular plus extracellular) histamine [S11]. All experiments were performed in triplicates. In control experiments performed with BM MNC, bardoxolone methyl and omaveloxolone (up to 10 μM) did not affect cell viability (>95% cells viable) after 30 minutes of incubation at 37°C as

determined by trypan blue exclusion using a Countess 3 Automated Cell Counter (Invitrogen, Carlsbad, CA, US).

Western Blot experiments

HMC-1, ROSA and MCPV-1 cells were incubated in control medium (co) or in medium containing various concentrations of drugs (bardoxolone methyl, omaveloxolone, midostaurin, avapritinib, nintedanib) as indicated. Thereafter, cells were harvested, lysed and Western blotting was performed using standard techniques [S6]. Primary antibodies against HO-1 (Enzo Life Sciences, Inc., Farmingdale, NY, US, rabbit, polyclonal, 1:1000), AKT (Cell Signaling Technology Inc., Danvers, MA, US, rabbit, polyclonal, 1:1000), p-AKT (Ser473) (Cell Signaling Technology Inc., Danvers, MA, US, rabbit, monoclonal, 1:1000), KIT (Santa Cruz Biotechnology, Santa Cruz CA, US, mouse, monoclonal, 1:1000), p-KIT (Tyr719) (Cell Signaling Technology Inc., Danvers, MA, US, rabbit, polyclonal, 1:500) were used. As loading controls mAb against actin (Santa Cruz Biotechnology, monoclonal, 1:1000), and β-tubulin (Santa Cruz Biotechnology, mouse, monoclonal, 1:2000) were applied. A list of the primary antibodies used is provided (Supplementary Table S3). Antibody reactivity was detected by applying secondary antibodies (sheep anti-mouse IgG or donkey anti-rabbit IgG; GE Healthcare, Buckinghamshire, UK) and ECL Plus Western blotting substrate (Thermo Scientific, Rockford, IL, US) [S6]. The percentage of viable cells was determined by trypan blue exclusion.

qPCR analysis

To study the effects of bardoxolone methyl and omaveloxolone on *KIT* and *AKT* mRNA expression levels, HMC-1.2 cells were kept in control medium or were treated with various concentrations of these drugs at 37°C for 1 to 24 h. In a separate set of experiments, qPCR was applied to determine mRNA expression levels of HO-1 after shRNA-induced knockdown. RNA was isolated from cell line cells using the RNeasy Mini Kit (Qiagen, Hilden, Germany). cDNA was synthesized using Moloney murine leukemia virus reverse transcriptase (Invitrogen, Carlsbad, CA, US), random primers, first strand buffer, dNTPs (100 mM), and RNasin (all from Invitrogen) according to the manufacturer's instructions. qPCR was performed as reported previously [S7] using primers shown in the Supplementary Table S4.

shRNA knockdown experiments

For knockdown of HMOX-1 (HO-1), hairpins in a microRNA-E (miR-E) backbone were cloned into the pRRL-based SGEP vector as described [S12]. Knockdown of *HMOX1* (heme oxygenase-1, HO-1) was achieved using three independent short hairpin RNA (shRNA) constructs. The guide sequences for each construct are shown in Supplementary Table S5. shRNA targeting Renilla luciferase (shRen.713) was used as a non-targeting control (NTC). For the production of recombinant vesicular stomatitis virus G glycoprotein (VSV-G) pseudotyped lentiviruses, the $CaPO_4$ method was used. In brief, 2 µg VSV-G-expressing envelope plasmid, 15.33 µg packaging plasmid ΔR8.91, and 13.33 µg transfer plasmid SGEP (containing the shRNAs of interest - HO-1 hairpin 1, 2, or 3, or a control shRNA targeting Renilla luciferase, Ren.713) were diluted in 900 µL ddH_2O. Then, 100 µL of 2.5 M $CaCl_2$ (Sigma-Aldrich, St. Louis, MO,

US) was added, vortexed, and incubated for 5 minutes at room temperature. Next, 1 mL HEPES-buffered saline (Sigma-Aldrich) was added under agitation, and the mixture was spread drop by drop onto approximately 50% confluent HEK293T cells (Thermo Fisher Scientific, Waltham, MA, US) in a 10-cm Petri dish. Cells were cultured at 37°C and 5% CO_2, and medium was changed after 24 h. After another 48 h of incubation, supernatants containing the viruses were harvested and centrifuged at 900× *g* for 5 minutes to remove cellular debris. For transduction, supernatants were immediately added to HMC-1.2 cells (1 mL supernatant per 1×10^6 cells), and cells were centrifuged at 900×*g* for 90 minutes in the presence of polybrene (8 µg/mL; Sigma-Aldrich). Cells were selected with 2 µg/mL puromycin (Sigma-Aldrich), and knockdown was confirmed by quantitative PCR (qPCR). Oligonucleotides were ordered from Integrated DNA Technologies (IDT, Coralville, IA, US). For competitive growth assays, GFP-positive HMC-1.2 cells, transduced with shRNA constructs targeting HO-1 (shHO-1; hairpins hp1, hp2, or hp3) and GFP-negative untransduced HMC-1.2 cells were co-cultured at an approximately equal ratio in DMSO control medium or in the presence of bardoxolone methyl (500 nM) for up to 28 days. The percentage of GFP-positive (shHO-1-transduced) and GFP-negative cells was determined by flow cytometry.

II. Supplementary References

S1. Butterfield JH, Weiler D, Dewald G, Gleich GJ. Establishment of an immature mast cell line from a patient with mast cell leukemia. Leuk Res. 1988;12(4):345-355.

S2. Saleh R, Wedeh G, Herrmann H, et al. A new human mast cell line expressing a functional IgE receptor converts to tumorigenic growth by KIT D816V transfection. Blood. 2014;124(1):111-120.

S3. Hoermann G, Blatt K, Greiner G, et al. CD52 is a molecular target in advanced systemic mastocytosis. FASEB J. 2014;28(8):3540-3551.

S4. Arock M, Wedeh G, Hoermann G, et al. Preclinical human models and emerging therapeutics for advanced systemic mastocytosis. Haematologica. 2018;103(11):1760-1771.

S5. Eisenwort G, Sadovnik I, Schwaab J, et al. Identification of a leukemia-initiating stem cell in human mast cell leukemia. Leukemia. 2019;33(11):2673-2684.

S6. Gleixner KV, Mayerhofer M, Cerny-Reiterer S, et al. KIT-D816V-independent oncogenic signaling in neoplastic cells in systemic mastocytosis: role of Lyn and Btk activation and disruption by dasatinib and bosutinib. Blood. 2011;118(7):1885-1898.

S7. Stefanzl G, Berger D, Cerny-Reiterer S, et al. The pan-BCL-2-blocker obatoclax (GX15-070) and the PI3-kinase/mTOR-inhibitor BEZ235 produce cooperative growth-inhibitory effects in ALL cells. Oncotarget. 2017; 8(40):67709–67722.

S8. Chou TC, Talalay P. Quantitative analysis of dose-effect relationships: the combined effects of multiple drugs or enzyme inhibitors. Adv Enzyme Regul. 1984;22:27-55.

S9. Zheng S, Wang W, Aldahdooh J, et al. SynergyFinder Plus: Toward Better Interpretation and Annotation of Drug Combination Screening Datasets. *Genomics* Proteomics Bioinformatics. 2022;20(3):587-596.

S10. Yadav B, Wennerberg K, Aittokallio T, Tang J. Searching for Drug Synergy in Complex Dose-Response Landscapes Using an Interaction Potency Model. *Comput* Struct Biotechnol J. 2015;13:504-513.

S11. Krauth MT, Mirkina I, Herrmann H, Baumgartner C, Kneidinger M, Valent P. Midostaurin (PKC412) inhibits immunoglobulin E-dependent activation and mediator release in human blood basophils and mast cells. Clin Exp Allergy. 2009;39(11):1711-1720.

S12. Fellmann C, Hoffmann T, Sridhar V, et al. An optimized microRNA backbone for effective single-copy RNAi. *Cell Rep.* 2013;5(6):1704–1713.

III. Supplementary Tables

Supplementary Table S1

Culture reagents and drugs used in this study

Compound	Dissolved in	Company
Dimethyl-sulfoxide (DMSO)	-	Merck (Darmstadt, Germany)
Iscove's modified Dulbecco's medium (IMDM)	-	Lonza (Verviers, Belgium)
Fetal calf serum (FCS)	-	Gibco life technologies (Gaithersburg, MD, US)
Penicillin	-	Lonza (Verviers, Belgium)
Streptomycin	-	Lonza (Verviers, Belgium)
Amphotericin	-	PAN Biotech (Aidenbach, Germany)
Alpha-thioglycerol	-	Sigma (St. Louis, MO, US)
DAPI	-	eBioscience (San Diego, CA, US)
^{3}H-thymidine	-	Perkin Elmer (Boston, MA, US)
Annexin V/APC	-	Biolegend (San Diego, CA, US)
Trypan Blue	-	Invitrogen (Carlsbad, CA, US)
Avapritinib	DMSO	Selleckchem (Houston, TX, US)
Midostaurin	DMSO	LC Laboratories (Woburn, MA, US)
Nintedanib	DMSO	Selleckchem (Houston, TX, US)
SMA-ZnPP	AquaBiDest	Faculty of Pharmaceutical Sciences, Sojo University, Kumamoto, Japan
Bardoxolone methyl (CDDO-Me)	DMSO	Med Chem Express LLC (NJ, US)
Omaveloxolone	DMSO	Med Chem Express LLC (NJ, US)
OB-24	DMSO	Med Chem Express LLC (NJ, US)

Abbreviations: AquaBiDest, bidistilled water; DAPI, 4′,6-diamidino-2-phenylindole.

Supplementary Table S2

Monoclonal antibodies used in flow cytometry experiments

Antigen	CD	Clone	Source	Isotype	Conjugate	Company
mIgG1	n.c.	MOPC-21	mouse	IgG1	PE	BD Biosciences (San Jose, CA, US)
mIgG1	n.c.	MOPC-21	mouse	IgG1	Alexa Fluor 647	BD Biosciences (San Jose, CA, US)
mIgG2a	n.c.	20102	mouse	IgG2a	PE	R&D Systems (Minneapolis, MN, US)
mIgG2b	n.c.	133303	mouse	IgG2b	PE	R&D Systems (Minneapolis, MN, US)
IL2-Rα	CD25	BC96	mouse	IgG1	APC-Cy7	Biolegend (San Diego, CA, US)
HPCA-1	CD34	581	mouse	IgG1	PE	BD Biosciences (San Jose, CA, US)
T10	CD38	HIT2	mouse	IgG1	APC	BD Biosciences (San Jose, CA, US)
LCA	CD45	2D1	mouse	IgG1	APC-H7	Biolegend (San Diego, CA, US)
Hermes-R	CD44	515	mouse	IgG1	PE	BD Biosciences (San Jose, CA, US)
LAMP-3	CD63	CLB-gran12	mouse	IgG1	PE	Immunotech (Marseille, France)
Transferrin-R	CD71	RI7217	mouse	IgG2a	PE	Biolegend (San Diego, CA, US)
APO-1, FAS	CD95	DX2	mouse	IgG1	PE	BD Biosciences (San José, CA, US)
SCF-R/KIT	CD117	104D2	mouse	IgG1	PE	BD Biosciences (San Jose, CA, US)
E-NPP3	CD203c	97A6	mouse	IgG1	PE	BD Biosciences (San Jose, CA, US)
SCF-R/KIT	CD117	104D2	mouse	IgG1	PE-Cy7	Invitrogen (Carlsbad, CA, US)

Abbreviations: mIgG, monoclonal immunoglobulin G; CD, cluster of differentiation; n.c., not yet clustered; IL-2Rα, interleukin-2 receptor α; HPCA-1, hematopoietic progenitor cell antigen-1; LCA, leukocyte common antigen; Hermes-R, hermes receptor; LAMP-3, lysosome-associated membrane protein 3; Transferrin-R, transferrin receptor; IgG, immunoglobulin G; PE, phycoerythrin; APC, allophycocyanin; APO-1, apoptosis antigen 1; SCF-R, stem cell factor receptor; E-NPP3, ectonucleotide pyrophosphatase/phosphodiesterase 3.

Supplementary Table S3

Primary antibodies used in Western blot experiments

Antibody	Order number	Host	Clonality	Dilution	Company
HO-1	ADI-SPA-896	rabbit	polyclonal	1:1000	Enzo Life Sciences, Inc. (Farmingdale, NY, US)
AKT	9272	rabbit	polyclonal	1:1000	Cell Signaling Technology Inc. (Danvers, MA, US)
pAKT (Ser473)	4060	rabbit	monoclonal	1:1000	Cell Signaling Technology Inc. (Danvers, MA, US)
KIT	sc-393910	mouse	monoclonal	1:1000	Santa Cruz Biotechnology (Santa Cruz, CA, US)
p-KIT (Tyr719)	3391	rabbit	polyclonal	1:500	Cell Signaling Technology Inc. (Danvers, MA, US)
Actin	sc-58673	mouse	monoclonal	1:1000	Santa Cruz, Biotechnology (Santa Cruz, CA, US)
β-tubulin	sc-53140	mouse	monoclonal	1:2000	Santa Cruz Biotechnology (Santa Cruz, CA, US)

Abbreviations: HO-1, heme oxygenase-1; AKT, protein kinase B; pAKT, phosphorylated protein kinase B; KIT, stem cell factor receptor; p-KIT, phosphorylated stem cell factor receptor.

Supplementary Table S4

Primers used for qPCR

Gene	Sequence
Human *HO-1*	5'-CAGGATTTGTCAGAGGCCCTGAAGG-3' (fwd)<br>5'-TGTGGTACAGGGAGGCCATCACC-3' (rev)
Human *KIT*	5'-GCGTTCTGCTCCTACTGCTTCG-3' (fwd)<br>5'-CCCTGGACTCACAGATGGTTGA-3' (rev)
Human *AKT*	5'-ACTGTCATCGAACGCACCTT-3' (fwd)<br>5'-CTGCTTCTTGAGGCCGTCA-3' (rev)
Human *GUSB*	5'-GGGCTTCGAGGAGCAGTGG-3' (fwd)<br>5'-GCTGGAGGGAACTGGCATGT-3' (rev)
Human *ABL1*	5'-TGTATGATTTTGTGGCCAGTGGAG-3' (fwd)<br>5'-GCCTAAGACCCGGAGCTTTTCA-3' (rev)

qPCR, quantitative polymerase chain reaction analysis; fwd, forward; rev, reverse.

Supplementary Table S5

shRNA guide sequences used for lentiviral knockdown of heme oxygenase-1 (HO-1)

**Target**	**shRNA**	**Guide sequence (5'-3')**
HMOX1 (HO-1)	#1	TTTGTGTAAAAATTGCCATGGC
HMOX1 (HO-1)	#2	TTATTTCACACAAAAGTTAGAC
HMOX1 (HO-1)	#3	TTGTGTAAAAATTGCCATGGCC
Renilla (control)	Ren.713	TAGATAAGCATTATAATTCCTA

Short hairpin RNA (shRNA) constructs (#1-#3) were used to knock down distinct regions of the human *HMOX1* transcript encoding heme oxygenase-1 (HO-1). shRNA targeting Renilla luciferase (shRen.713) was used as a non-targeting control.

Supplementary Table S6

Cooperative (synergistic or additive) effects between triterpenoids and other drugs (KIT D816V targeting TKI / SMA-ZnPP/ OB-24) on proliferation of human MC lines as assessed by ^{3}H-thymidine uptake experiments

Cell line	D1 + D2 combination	Range D1 (nM)	Range D2 (nM)	Ratio	CI value	Synergy scores	
						ZIP	HSA
HMC-1.1	Bardoxolone methyl + SMA-ZnPP	200-320	3800-6000	1:19	CI < 1 for all conc. tested	49.59	37.33
HMC-1.2	Bardoxolone methyl + SMA-ZnPP	200-320	3800-6000	1:19	CI < 1 for all conc. tested	29.43	29.46
HMC-1.1	Omaveloxolone + SMA-ZnPP	200-320	3800-6000	1:19	not applicable	50.16	38.74
HMC-1.2	Omaveloxolone + SMA-ZnPP	200-320	3800-6000	1:19	CI < 1 for all conc. tested	49.7	34.13
MCPV-1.3	Bardoxolone methyl + SMA-ZnPP	300-390	4100-5330	3:41	not applicable	7.26	14.26
MCPV-1.4	Bardoxolone methyl + SMA-ZnPP	300-390	4100-5330	3:41	CI < 1 for all conc. ≥ "315+4300 nM"	9.97	19.56
HMC-1.1	Bardoxolone methyl + midostaurin	200-350	200-350	1:1	CI < 1 for all conc. ≥ "275+275 nM"	5.09	16.06
HMC-1.2	Bardoxolone methyl + midostaurin	175-325	87.5-162.5	2:1	CI < 1 for all conc. tested	-11.9	3.38
MCPV-1.1	Bardoxolone methyl + midostaurin	200-400	200-400	1:1	CI 1-2 for all conc. tested	0.56	8.82
MCPV-1.2	Bardoxolone methyl + midostaurin	200-400	200-400	1:1	CI 1-2 for all conc. tested	-1.02	10.95
MCPV-1.3	Bardoxolone methyl + midostaurin	200-400	200-400	1:1	CI < 1 for all conc. tested	9.12	6.47
MCPV-1.4	Bardoxolone methyl + midostaurin	200-400	200-400	1:1	CI 1-2 for all conc. tested	4.75	7.23
HMC-1.1	Omaveloxolone + midostaurin	200-350	200-350	1:1	CI < 1 for all conc. tested	10.72	20.21
HMC-1.2	Omaveloxolone + midostaurin	150-300	75-150	2:1	not applicable	44.19	33.28
MCPV-1.1	Omaveloxolone + midostaurin	250-400	250-400	1:1	not applicable	30.21	30.95
MCPV-1.2	Omaveloxolone + midostaurin	250-400	250-400	1:1	CI < 1 for all conc. ≥ "275+275 nM"	27.78	31.06
MCPV-1.3	Omaveloxolone + midostaurin	250-400	250-400	1:1	CI < 1 for all conc. tested	26.57	33.85
MCPV-1.4	Omaveloxolone + midostaurin	250-400	250-400	1:1	CI 1-2 for all conc. tested	24.14	24.14
HMC-1.1	Bardoxolone methyl + avapritinib	200-260	200-260	1:1	CI < 1 for all conc. tested	32.49	41.77
HMC-1.2	Bardoxolone methyl + avapritinib	200-300	100-150	2:1	CI < 1 for all conc. tested	36.22	22.33

HMC-1.1	Omaveloxolone + avapritinib	200-260	200-260	1:1	CI < 1 for all conc. tested	28.27	36.67
HMC-1.2	Omaveloxolone + avapritinib	200-320	100-160	2:1	CI < 1 for all conc. tested	29.62	33.02
MCPV-1.1	Bardoxolone methyl + avapritinib	300-390	250-325	30:25	CI > 1 for all conc. tested	12.36	30.61
HMC-1.1	Bardoxolone methyl + nintedanib	280-340	6.6-8	17:0.4	CI < 1 for all conc. tested	22.74	36.83
HMC-1.2	Bardoxolone methyl + nintedanib	280-340	280-340	1:1	not applicable	12.57	30.67
HMC-1.1	Omaveloxolone + nintedanib	200-320	5-7.5	100:2.5	CI < 1 for all conc. tested	31.19	39.18
HMC-1.2	Omaveloxolone + nintedanib	200-320	200-320	1:1	not applicable	58.32	32.62
HMC-1.2	Bardoxolone methyl + OB-24	200-320	12500-20000	2:125	not applicable	38.19	39.37
HMC-1.2	Omaveloxolone + OB-24	200-320	12500-20000	2:125	not applicable	36.94	21.69
Pat. #4	Bardoxolone methyl + midostaurin	175-325	175-325	1:1	CI < 1 for all conc. tested	-8.98	5.15
Pat. #5	Bardoxolone methyl + midostaurin	175-325	175-325	1:1	CI < 1 for "325+325 nM"	1.43	11.06
Pat. #11.3	Bardoxolone methyl + midostaurin	87.5-162.5	175-325	1:2	CI < 1 for all conc. ≥ "112.5+225 nM"	1.17	16.52
Pat. #15	Omaveloxolone + midostaurin	175-325	175-325	1:1	CI < 1 for all conc. tested	8.56	10.87
Pat. #19.2	Omaveloxolone + midostaurin	175-325	175-325	1:1	not applicable	-4.99	7.99
Pat. #23	Bardoxolone methyl + midostaurin	175-325	175-325	1:1	not applicable	-6.66	9.85

For evaluation of potential cooperative (synergistic or additive) drug-effects, cell lines or primary cells were incubated in various two-drug-combinations (as described in the main document) each involving a triterpenoid (bardoxolone methyl or omaveloxolone) and a KIT D816V-TKI (midostaurin, avapritinib or nintedanib) or an HO-1 inhibitor (SMA-ZnPP or OB-24) at a fixed ratio. Proliferation was determined by measuring uptake of ^{3}H-thymidine. Drug interaction-types were determined by 2 different methods. Combination index (CI) values were determined using CalcuSyn software. A CI of <1 indicates a synergistic effect, whereas a CI of >1 indicates antagonism. Furthermore, the type of drug combination effects was analyzed by calculating synergy scores ((zero interaction potency, ZIP and highest single agent (HSA) synergy score)) by SynergyFinder Plus software. A synergy score >10 stands for synergism, a synergy score between -10 and 10 for an additive effect and a synergy score <-10 stands for antagonism. Abbreviations: D: drug; nM: nanomolar; conc.: concentrations; CI: combination index; ZIP: zero interaction potency; HSA: highest single agent synergy score [S8-S10]. Patient numbers refer to Table 1 in the main document.

IV. Supplementary Figures

Figure S1: Effects of triterpenoids in primary neoplastic and normal BM cells

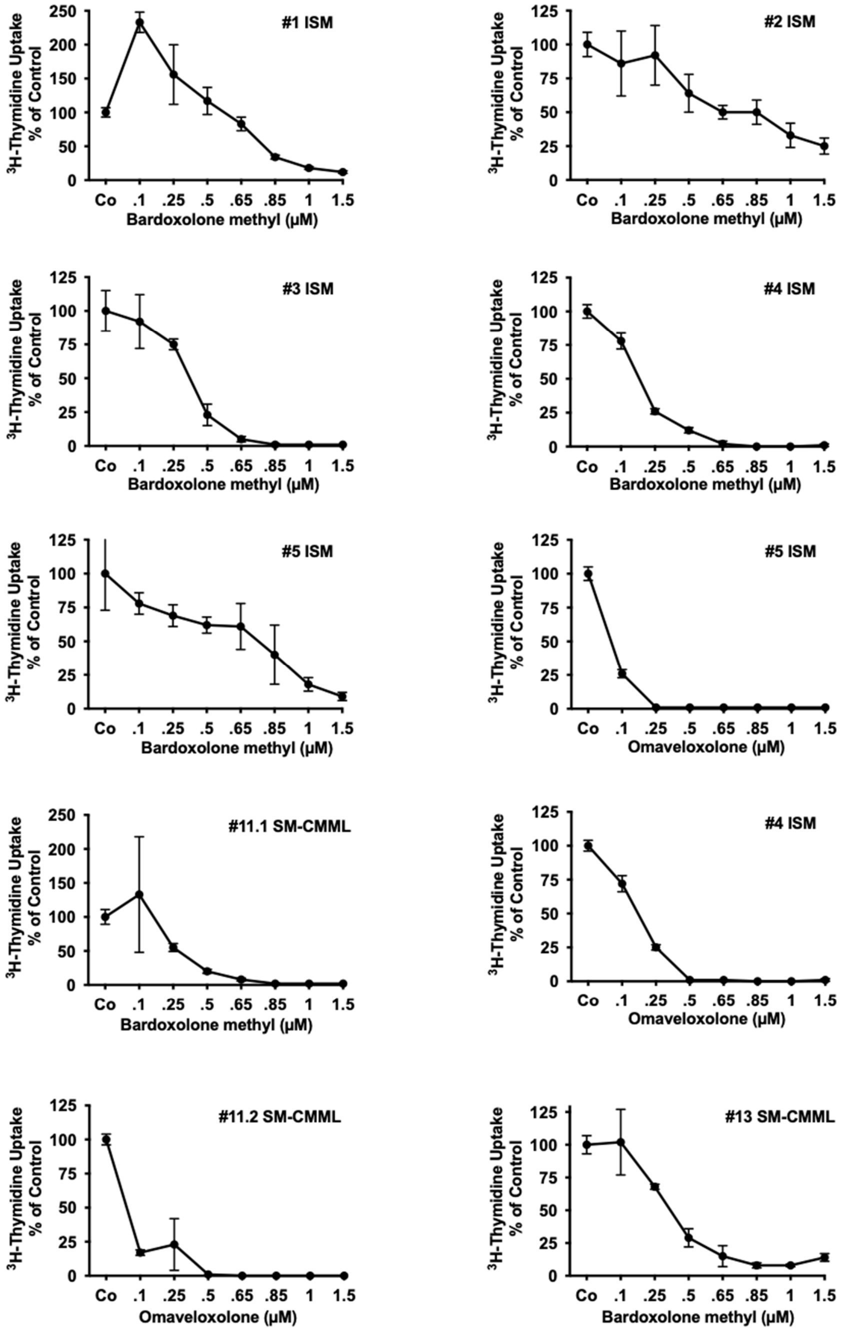

John et al, Supplementary Figure S1A

Bardoxolone methyl and omaveloxolone inhibit the proliferation of primary neoplastic MC

Primary cells isolated from patients with various types of systemic mastocytosis (SM) were incubated in control medium (Co) or various concentrations of bardoxolone methyl or omaveloxolone as indicated at 37°C for 48 hours (h). Then, proliferation was measured by assessing ^{3}H-thymidine uptake. Results are expressed in percent of control and represent the mean±S.D. of triplicates. Patient numbers refer to Table 1 of the main manuscript.

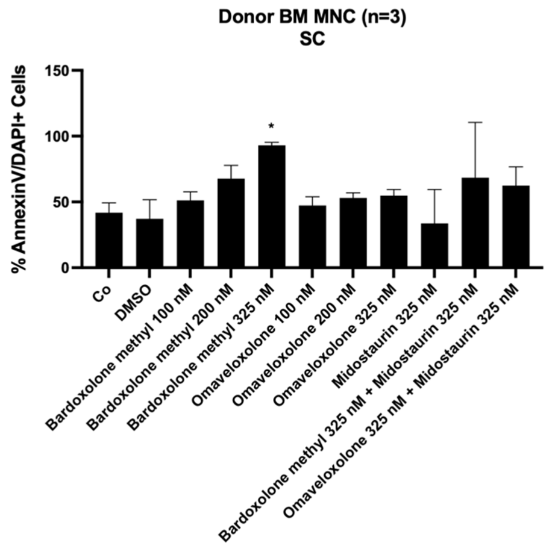

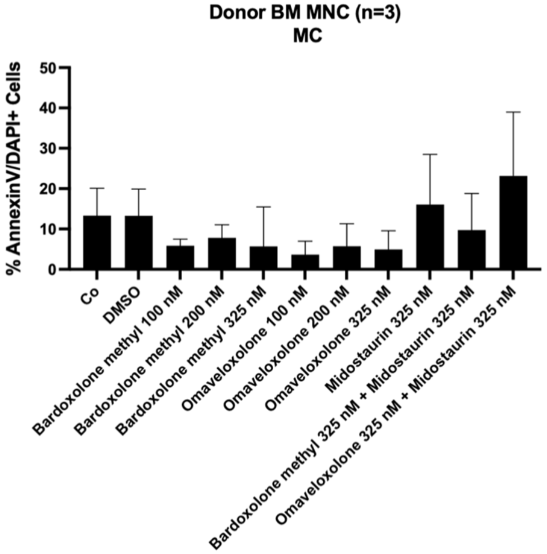

John et al, Supplementary Figure S1B

Effects of triterpenoids and midostaurin on viability of healthy BM stem cells and mast cells

Bone marrow (BM) mononuclear cells (MNC) isolated from healthy donors (n=3) were incubated in control medium (Co), DMSO, bardoxolone methyl (100-325 nM), omaveloxolone (100-325 nM), midostaurin (325 nM) or a combination of bardoxolone methyl/omaveloxolone and midostaurin, as indicated at 37°C for 48 hours. For apoptosis analysis, the percentage of Annexin V/DAPI-positive cells was determined in the hematopoietic progenitor and stem cells (SC: CD45+, CD34+, CD38-), and mast cells (MC, CD45+, CD117+, CD34-) gated by multicolor flow cytometry. Statistical significance was determined by one-way ANOVA with Bonferroni's correction for multiple comparisons; * $p<0.05$ compared to control.

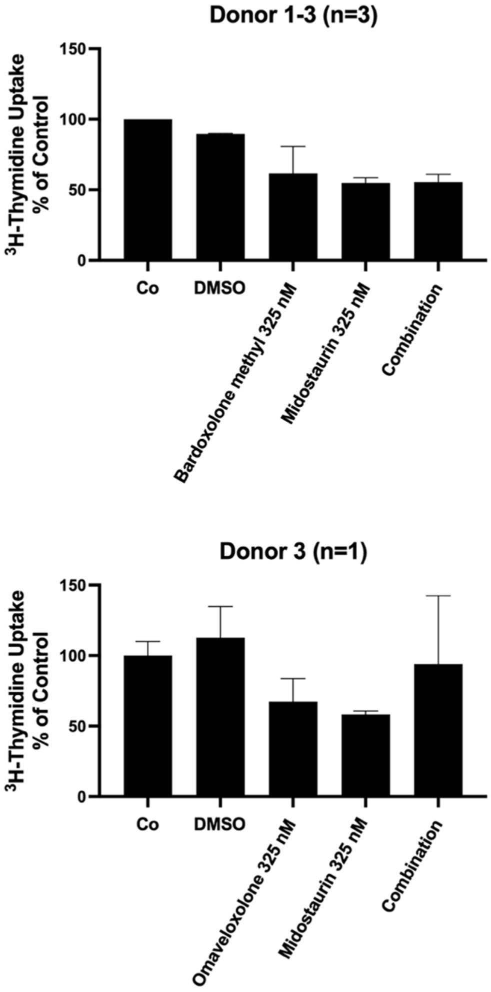

John et al, Supplementary Figure S1C

Effects of triterpenoids and midostaurin on proliferation of healthy BM MNC

Bone marrow (BM) mononuclear cells (MNC) isolated from healthy donors (n=3) were incubated in control medium (Co), DMSO, bardoxolone methyl (325 nM), omaveloxolone (325 nM), midostaurin (325 nM) or a combination of bardoxolone methyl/omaveloxolone and midostaurin, as indicated at 37°C for 48 hours. Then, proliferation was measured by assessing ^{3}H-thymidine uptake. Results show the percentage of ^{3}H–thymidine uptake compared with medium control and represent the mean±S.D. of 3 independent experiments (bardoxolone methyl; n=3) or triplicates (omaveloxolone; n=1).

Figure S2: Triterpenoid effects on expression of surface markers in neoplastic MC

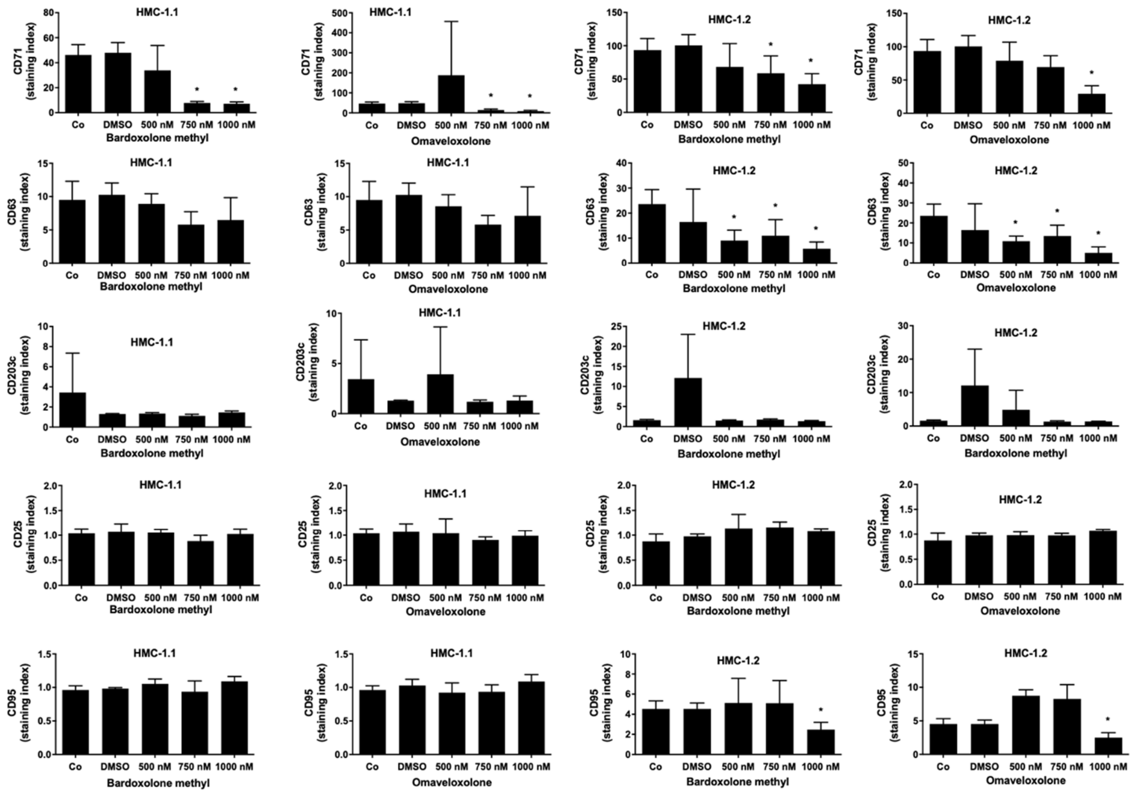

John et al, Supplementary Figure S2

Growth-inhibitory effects of bardoxolone methyl and omaveloxolone in neoplastic MC are accompanied by inhibition of relevant cell surface proteins in MCL-like cell lines HMC-1.1 and HMC-1.2 cells were incubated in control medium (Co), DMSO control medium or medium containing various concentrations of bardoxolone methyl or omaveloxolone, as indicated at 37°C for 24 hours. Then, surface expression of CD25, CD63, CD71, CD95 and CD203c was determined by flow cytometry using monoclonal antibodies (mAb) against indicated antigens. Staining index (SI) as defined by median fluorescence intensity was obtained with target mAb divided by the isotype-matched control mAb. Results represent the mean±S.D. of 3 experiments. * $p<0.05$ compared to control, using one-way ANOVA with Bonferroni's correction for multiple comparisons.

Figure S3: Effect of bardoxolone methyl and omaveloxolone on AKT and KIT signaling

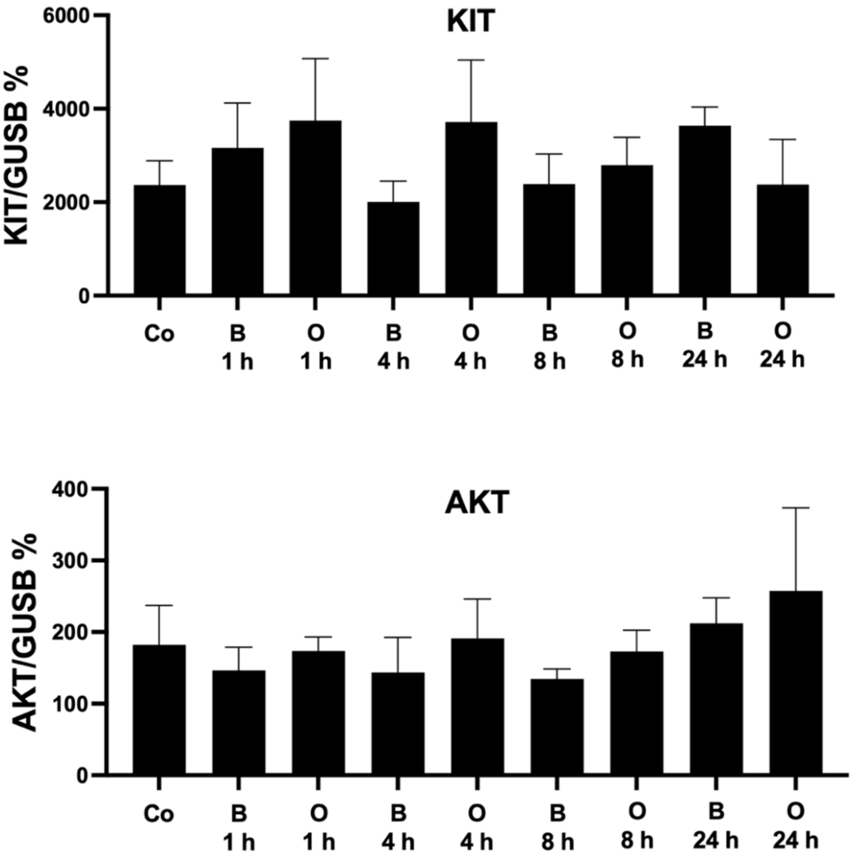

John et al, Supplementary Figure S3A

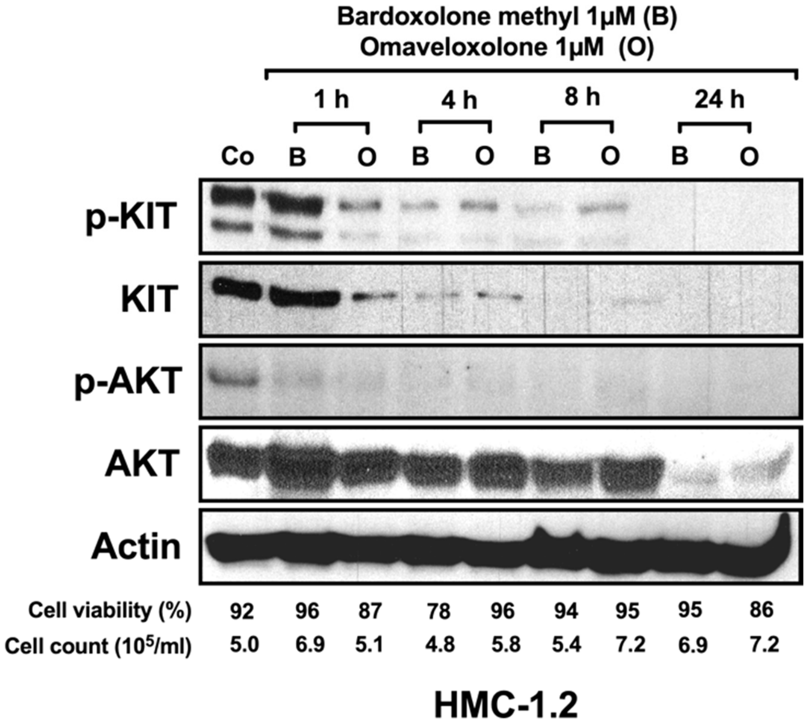

John et al, Supplementary Figure S3B

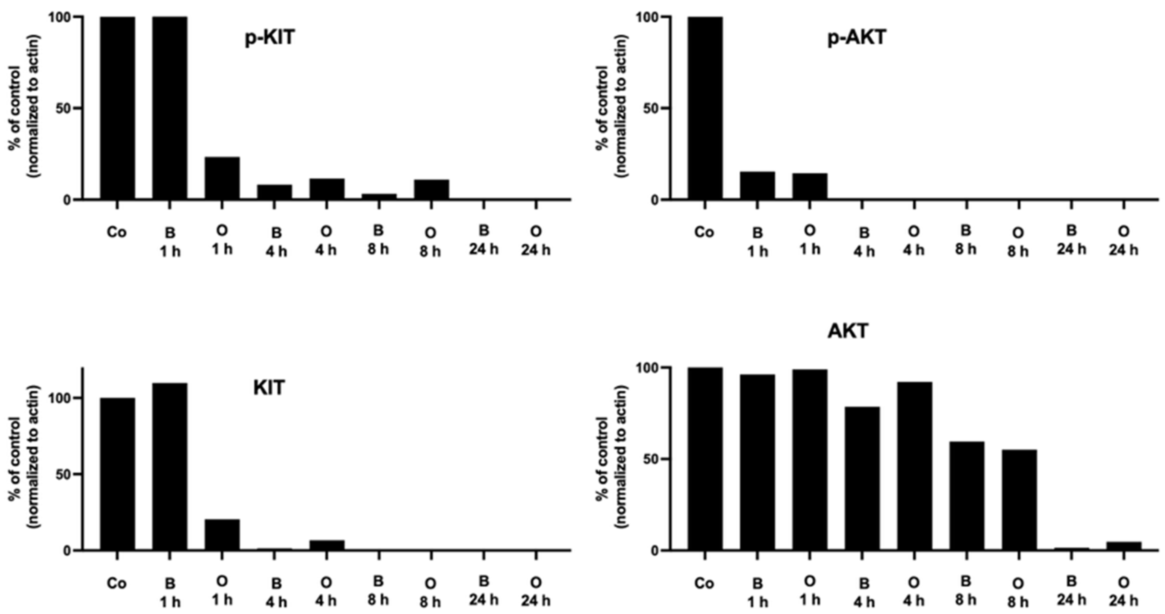

John et al, Supplementary Figure S3C

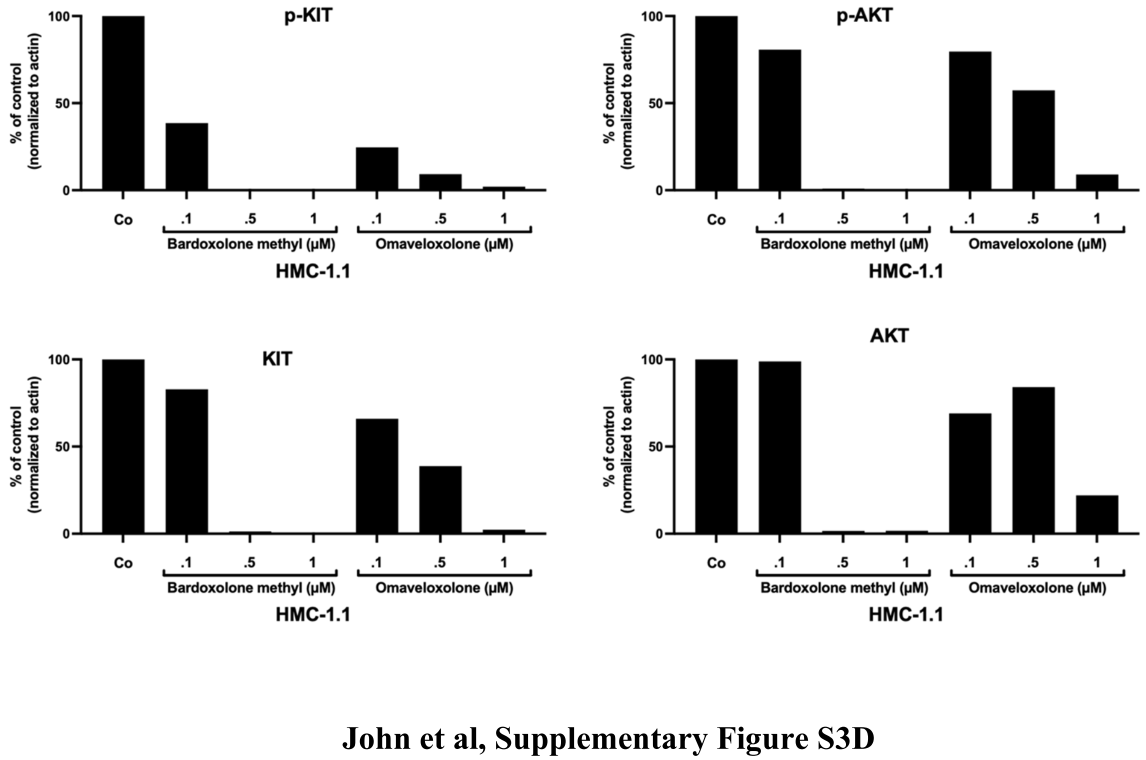

John et al, Supplementary Figure S3D

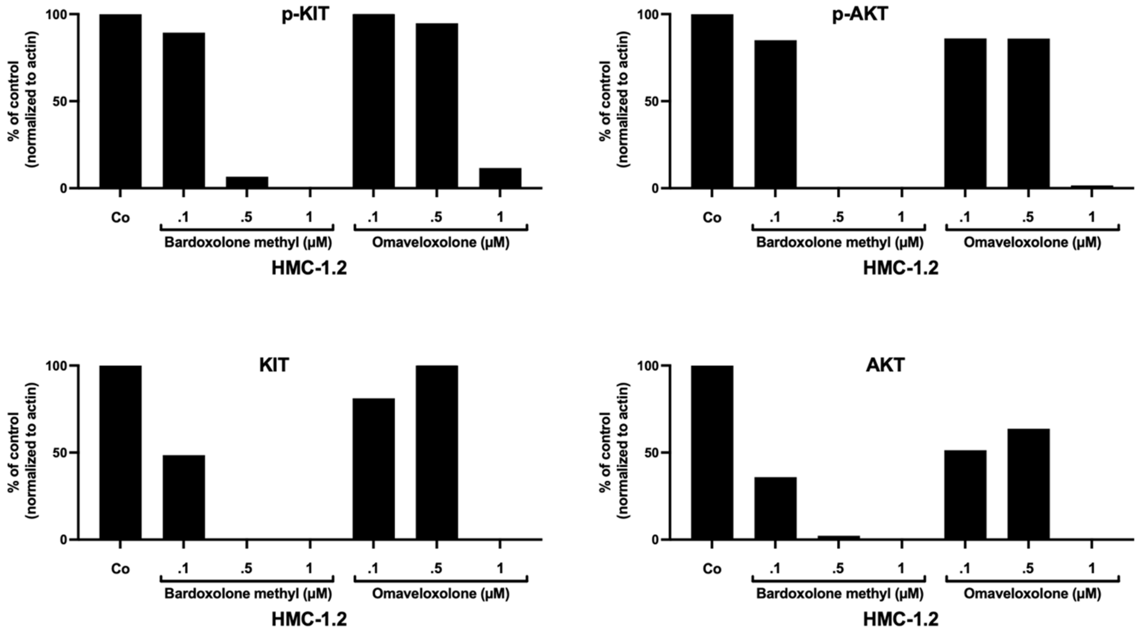

John et al, Supplementary Figure S3E

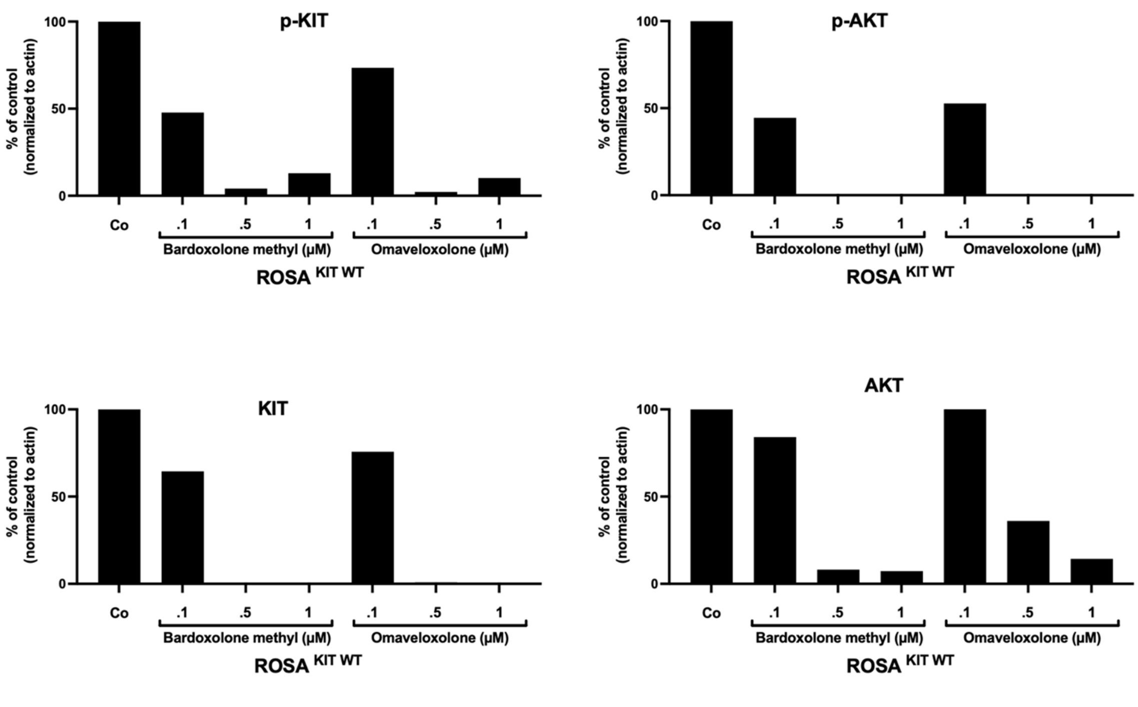

John et al, Supplementary Figure S3F

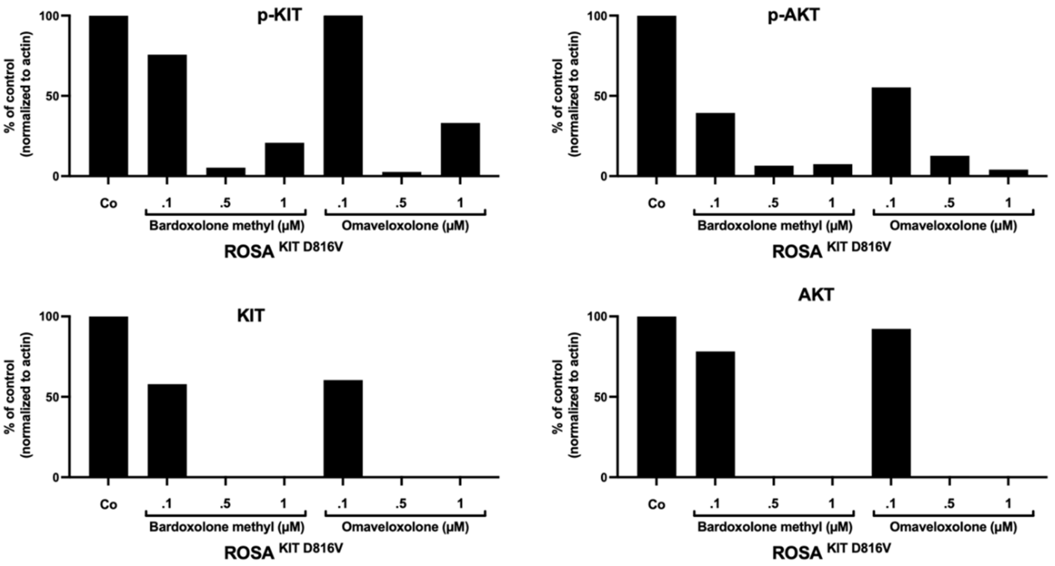

John et al, Supplementary Figure S3G

Effect of bardoxolone methyl and omaveloxolone on AKT and KIT signaling and number of viable cells in HMC-1.2 cells

(A) HMC-1.2 cells were incubated with DMSO control medium (Co), bardoxolone methyl (B; 1 µM), or omaveloxolone (O; 1 µM) for 1, 4, 8, or 24 hours (h). Relative mRNA expression of *KIT* and *AKT* was determined by qPCR and normalized to *GUS*B; values are expressed as a percentage of *GUSB* expression. Data are shown as mean ±SEM (n = 3; independent experiments). **(B)** HMC-1.2 cells were incubated with DMSO control medium (Co), bardoxolone methyl (B; 1 µM), or omaveloxolone (O; 1 µM) for 1, 4, 8, or 24 h. At each time point whole-cell lysates were analyzed by Western blotting for phosphorylated KIT (p-KIT), total KIT, phosphorylated AKT (p-AKT), total AKT, and actin (loading control). Cell viability (%) and cell counts ($\times 10^5$/mL) at the time of harvest were determined by trypan blue exclusion using a Countess 3 Automated Cell Counter (Invitrogen) and are indicated below the blot. Data are representative of one typical experiment. **(C)** Densitometric quantification of band intensities was performed using ImageJ (Fiji). For each lane, band intensity was normalized to the corresponding actin signal and expressed as a percentage of the untreated control (Co = 100%). Bars are shown in the order of the corresponding blot in (B). Results are shown for one representative experiment. Both compounds suppressed p-KIT and p-AKT within 1–4 h, followed by inhibition of total KIT protein expression, while total AKT expression declined over 24 h. **(D-G)** Densitometric quantification of band intensities performed using ImageJ (Fiji). Band intensity of each lane was normalized to the corresponding actin signal and expressed as a percentage of the untreated control (Co=100%). Bars are arranged in the same lane order as the corresponding blot in Figure 3 in the main manuscript: HMC-1.1 (D), HMC-1.2 cells (E), ROSA$^{KIT\ WT}$ (F), ROSA$^{KIT\ D816V}$ (G). Data are shown for one representative experiment per cell line.

Figure S4: shRNA-mediated knockdown of HO-1 in HMC-1.2 cells

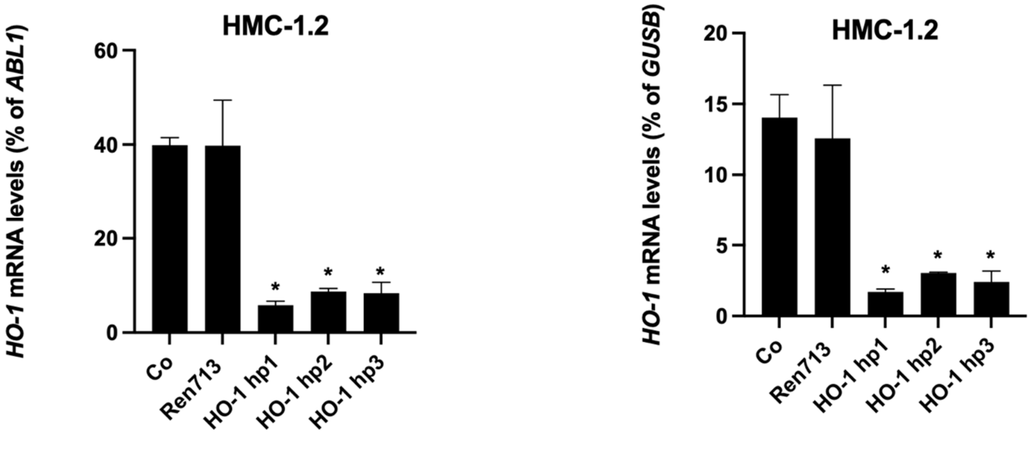

John et al, Supplementary Figure S4A

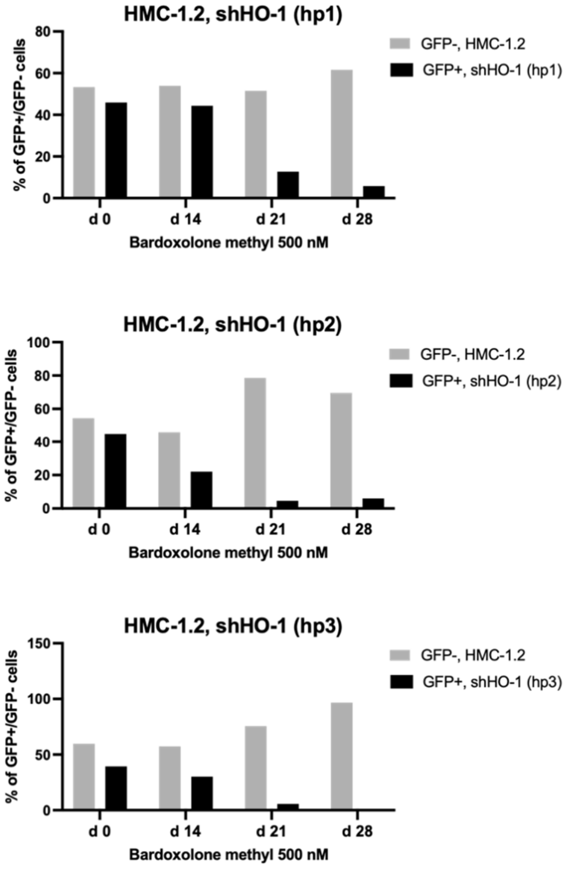

John et al, Supplementary Figure S4B

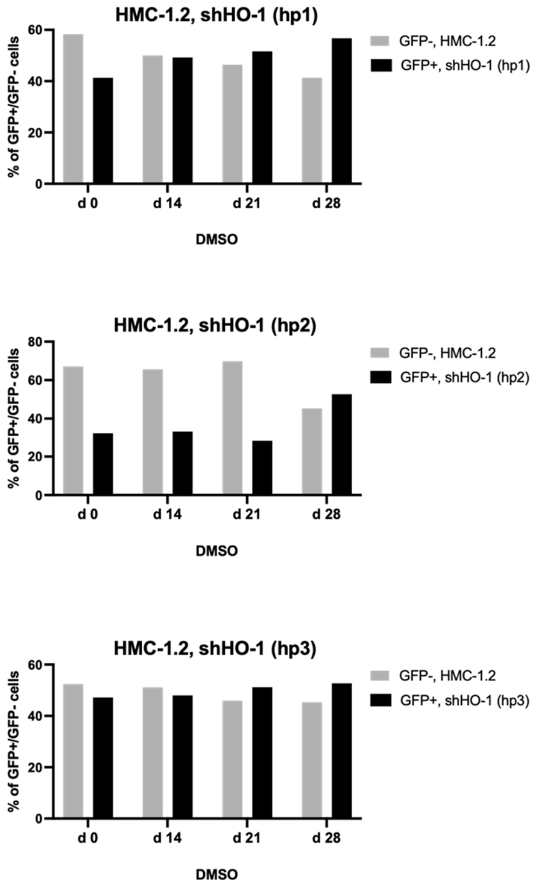

John et al, Supplementary Figure S4C

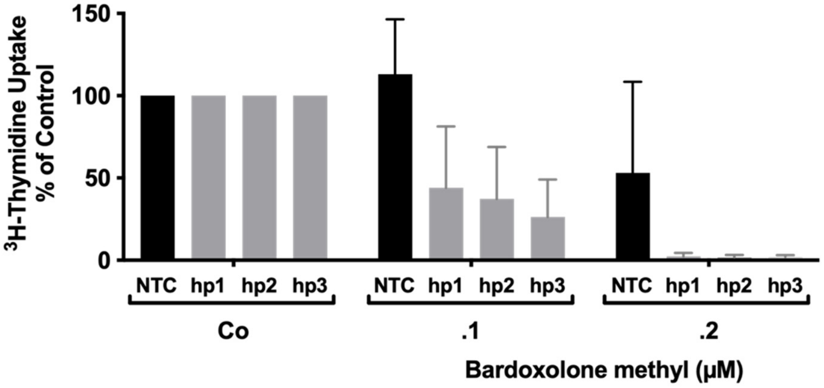

John et al, Supplementary Figure S4D

Effects of HO-1 knockdown on triterpenoid sensitivity in HMC-1.2 cells

(A) Knockdown efficiency of HO-1 shRNA constructs in HMC-1.2 cells. Untreated HMC-1.2 cells and HMC-1.2 cells expressing a non-targeting control shRNA (Ren713), or shRNA constructs targeting HO-1 (HO-1 hp1, hp2, hp3) were analyzed for *HO-1* mRNA expression by quantitative PCR (qPCR). HO-1 mRNA expression levels normalized to *ABL1* mRNA levels (left panel) and *HO-1* mRNA levels normalized to *GUSB* mRNA levels (right panel) are shown. Results represent the mean±S.D. of 3 independent experiments. Statistical analysis was performed using one-way ANOVA with Bonferroni's correction for multiple comparisons (*$p<0.05$ compared to control). **(B)** Knockdown of HO-1 sensitizes HMC-1.2 cells to bardoxolone methyl in a competitive growth assay. HMC-1.2 cells were transduced with shRNA constructs targeting HO-1 (shHO-1; hairpins hp1, hp2, or hp3) or left untransduced, mixed at an approximately equal ratio with untransduced GFP-negative HMC-1.2 cells, and cultured with bardoxolone methyl (500 nM) for up to 28 days. The percentage of GFP-positive (shHO-1-transduced) and GFP-negative (untransduced) cells was determined by flow cytometry at the indicated time points (day 0, 14, 21, and 28). Results show the relative proportion of GFP+ and GFP− cells within the culture and represent one typical experiment. **(C)** HO-1 knockdown does not impair the growth of HMC-1.2 cells in the absence of bardoxolone methyl. HMC-1.2 cells were transduced with shRNA constructs targeting HO-1 (shHO-1; hairpins hp1, hp2, or hp3) or left untransduced, mixed at an approximately equal ratio with untransduced GFP-negative HMC-1.2 cells, and cultured in control medium or medium containing DMSO or for up to 28 days. The percentage of GFP-positive (shHO-1-transduced) and GFP-negative (untransduced) cells was determined by flow cytometry at the indicated time points (day 0, 14, 21, and 28). Results are shown for one representative experiment per condition and hairpin construct. **(D)** HMC-1.2 cells expressing a non-targeting control shRNA (NTC) or one of three shRNAs targeting HO-1 (heme oxygenase-1; hp1, hp2, hp3) were incubated in control medium (Co) or 0.1 µM or 0.2 µM bardoxolone methyl as indicated at 37 °C for 48 hours. Then, proliferation was measured by assessing ^{3}H-thymidine incorporation. Results are expressed in percent of control and represent the mean±SD of 3 independent experiments.

Figure S5: HO-1 blockade sensitizes MC against treatment with triterpenoids

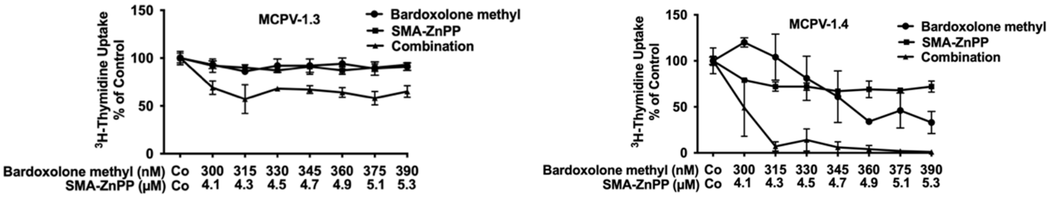

John et al, Supplementary Figure S5A

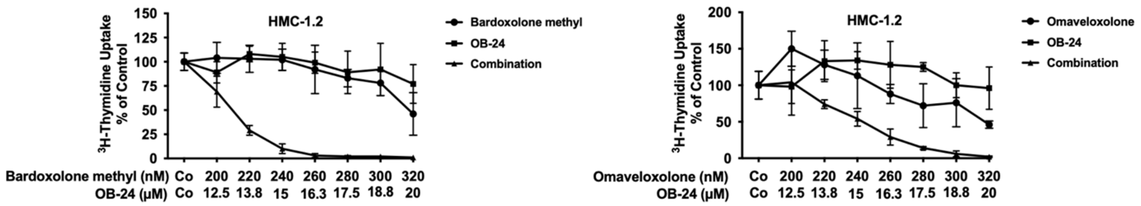

John et al, Supplementary Figure S5B

HO-1 blockade sensitizes neoplastic MC against treatment with triterpenoids

(A) Effects of the HO-1 inhibitor SMA-ZnPP in human MC lines. MCPV-1 cells were incubated in control medium (Co) or medium containing either bardoxolone methyl/omaveloxolone or the HO-1 inhibitor SMA-ZnPP alone, or a combination of both drugs (at fixed ratio) for 48 hours (h). Then, proliferation was measured by assessing ^{3}H-thymidine uptake. Results show the percentage of ^{3}H–thymidine uptake compared with medium control and represent the mean±S.D. of triplicates from 1 typical experiment. At least two additional experiments with corresponding results were performed for all combinations shown. **(B)** Bardoxolone methyl and omaveloxolone produce cooperative growth-inhibitory effects with OB-24. HMC-1.1 and HMC-1.2 cells were incubated in control medium (Co) or medium containing either bardoxolone methyl, omaveloxolone, or OB-24 alone, or a combination (at fixed ratio) of both drugs (at the concentrations indicated) for 48 h. Then, proliferation was measured by assessing ^{3}H-thymidine uptake. Results show the percentage of ^{3}H–thymidine uptake compared

with medium control and represent the mean±S.D. of triplicates from 1 typical experiment. At least two additional experiments with corresponding results were performed for all combinations shown.

Figure S6: Defining combinatorial effects by CalcuSyn software

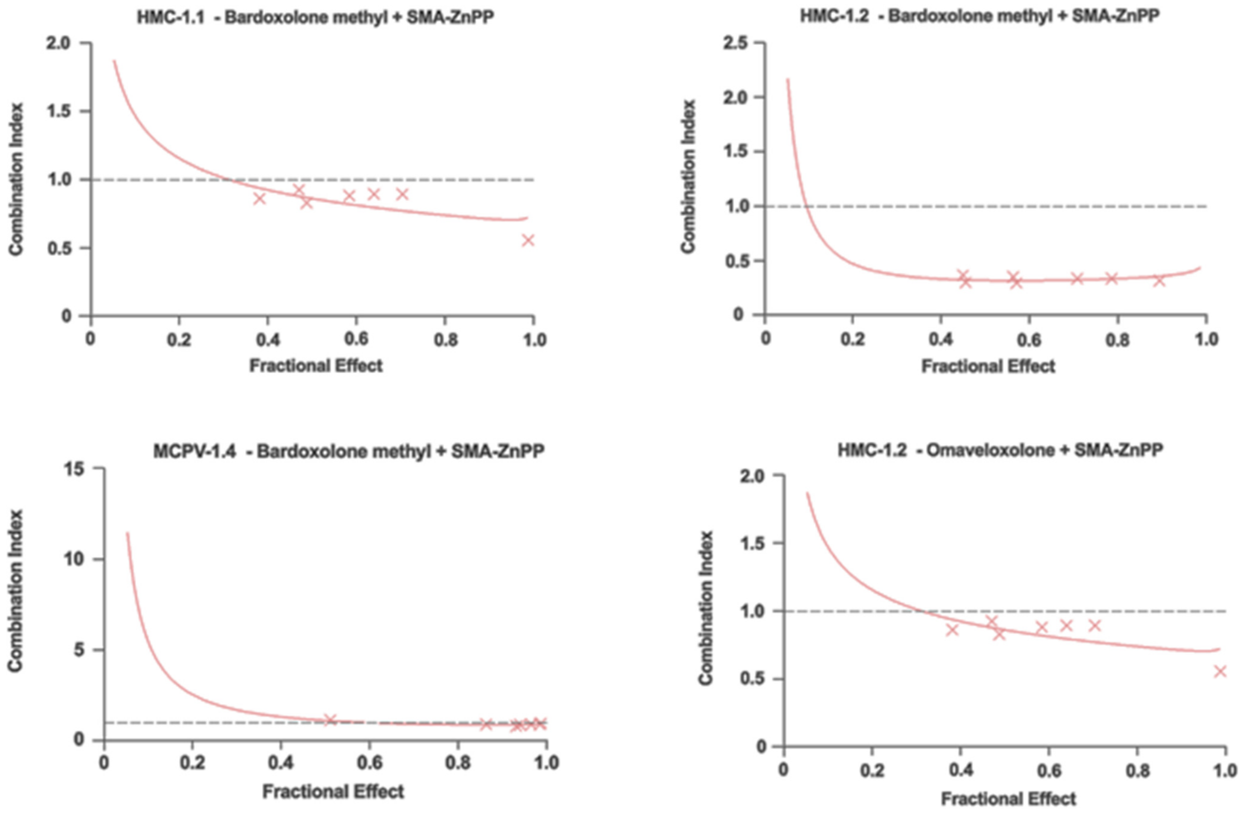

John et al, Supplementary Figure S6A

Defining the combinatorial effects of bardoxolone methyl or omaveloxolone and the HO-1 inhibitor SMA-ZnPP on growth of HMC-1 cells by CalcuSyn software
HMC-1.1 and HMC-1.2 cells were kept in control medium or in various concentrations of bardoxolone methyl (or omaveloxolone) and SMA-ZnPP alone or in combination at a fixed ratio at 37°C for 48 hours. Thereafter, cell proliferation was determined by measuring ^{3}H-thymidine uptake as shown in Figure S5A and in Figure 5C of the main document. The nature of drug interaction (additive versus synergistic) was determined for each experiment by calculating combination index (CI) values using CalcuSyn software. Resulting fractional effects and CI values are shown. A CI value of 1 indicates an additive effect, whereas CI values below 1 indicate synergistic drug effects.

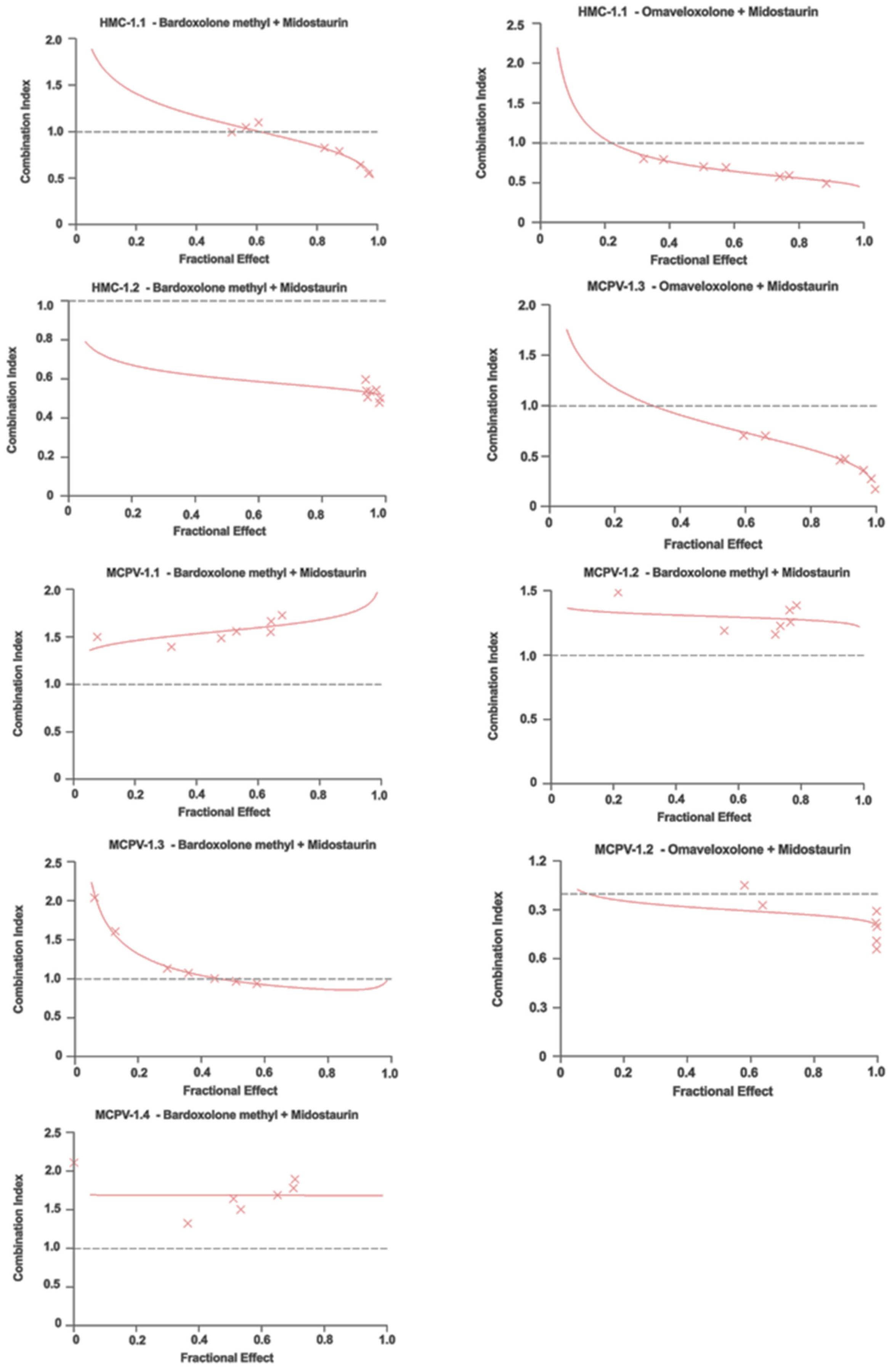
HMC-1.1 - Bardoxolone methyl + Midostaurin
HMC-1.1 - Omaveloxolone + Midostaurin
HMC-1.2 - Bardoxolone methyl + Midostaurin
MCPV-1.3 - Omaveloxolone + Midostaurin
MCPV-1.1 - Bardoxolone methyl + Midostaurin
MCPV-1.2 - Bardoxolone methyl + Midostaurin
MCPV-1.3 - Bardoxolone methyl + Midostaurin
MCPV-1.2 - Omaveloxolone + Midostaurin
MCPV-1.4 - Bardoxolone methyl + Midostaurin
Combination Index
Fractional Effect

John et al, Supplementary Figure S6B

Cooperative effects of bardoxolone methyl or omaveloxolone and the KIT D816V inhibitor midostaurin in MCL- like cell lines as determined by CalcuSyn software
HMC-1.1, HMC-1.2 and various MCPV-1 subclone cells were kept in control medium or in various concentrations of bardoxolone methyl (or omaveloxolone) and midostaurin alone or a combination of both drugs at a fixed ratio at 37°C for 48 hours. Thereafter, cell proliferation was determined by measuring ^{3}H-thymidine uptake as shown in Figures S6E and 6A of the main document. The nature of drug interaction (additive versus synergistic) was determined for each experiment by calculating combination index (CI) values using CalcuSyn software. Resulting fractional effects and CI values are shown. A CI value of 1 indicates an additive effect, whereas CI values below 1 indicate synergistic drug effects.

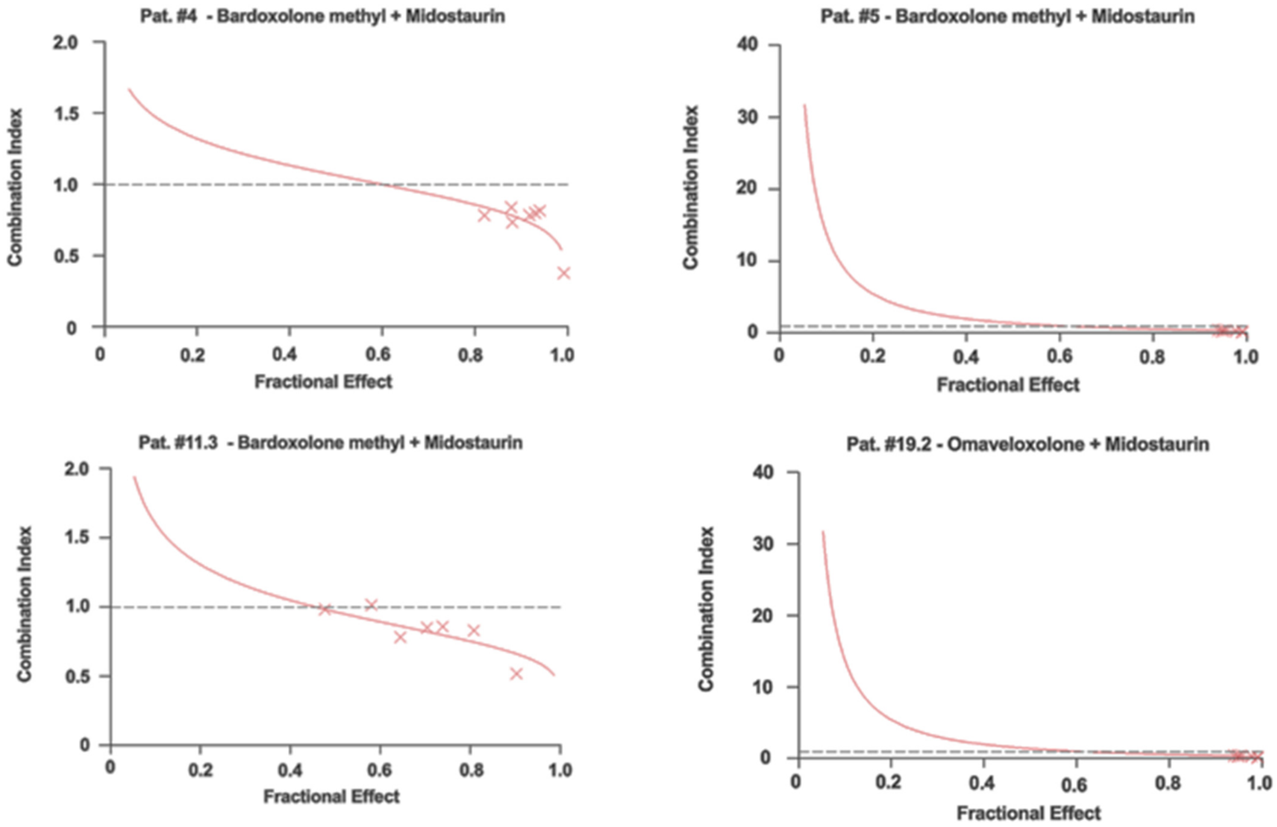

John et al, Supplementary Figure S6C

Defining the combinatorial effects of bardoxolone methyl or omaveloxolone and midostaurin in primary neoplastic mast cells as determined by CalcuSyn software Primary neoplastic MC isolated from BM of patients (patient numbers refer to Table 1) with ISM or advSM were kept in control medium or in various concentrations of bardoxolone methyl (or omaveloxolone) and midostaurin or a combination of both drugs at a fixed ratio at 37°C for 48 hours. Then, cell proliferation was determined by measuring ^{3}H-thymidine uptake as shown in Figure 6B in the main document. The nature of drug interaction (additive versus synergistic) was determined for each experiment by calculating combination index (CI) values using CalcuSyn software. Resulting fractional effects and CI values are shown. A CI value of 1 indicates an additive effect, whereas CI values below 1 indicate synergistic drug effects.

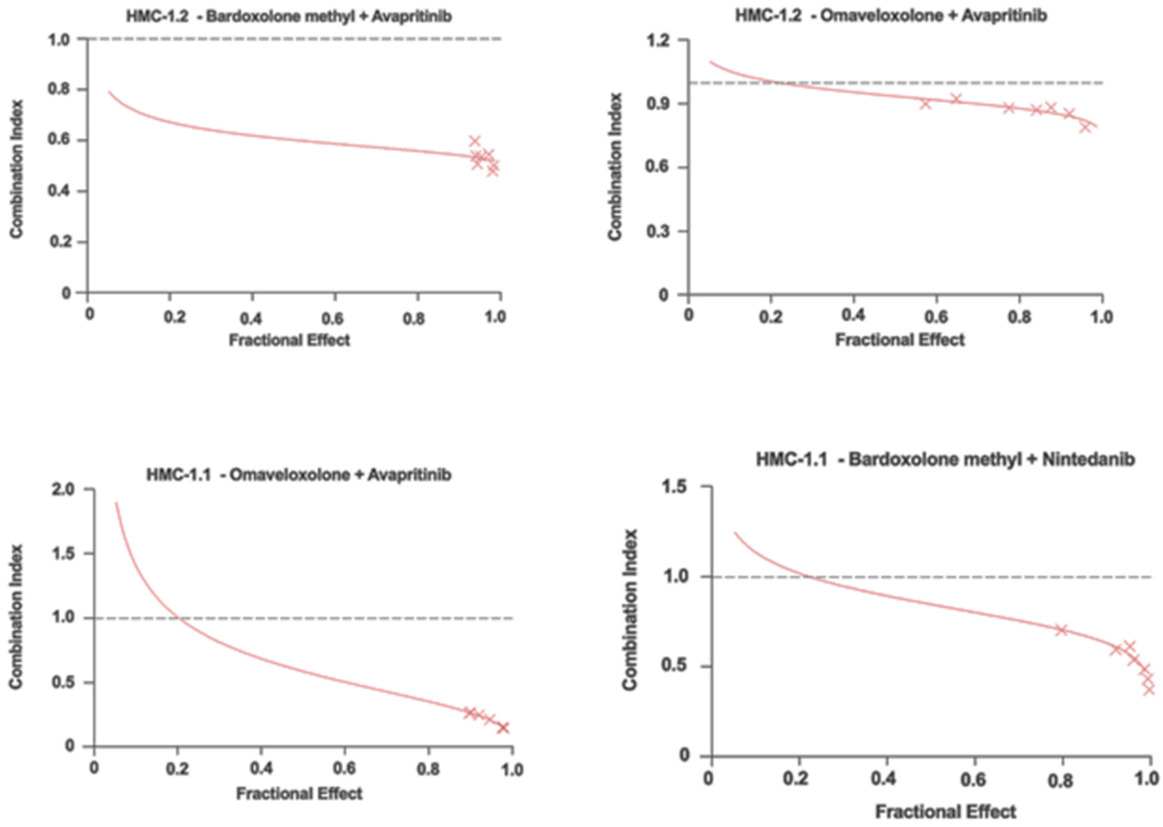

John et al, Supplementary Figure S6D

Defining the combinatorial effects of bardoxolone methyl or omaveloxolone and KIT D816V inhibitors on MCL-like cell lines as determined by CalcuSyn software HMC-1.1 and HMC-1.2 cells were kept in control medium or in various concentrations of bardoxolone methyl (or omaveloxolone) alone or together with one of the KIT D816V-targeting drugs (avapritinib or nintedanib) at a fixed ratio of drug concentrations at 37°C for 48 hours. Thereafter, cell proliferation was determined by measuring ^{3}H-thymidine uptake – primary results obtained with these drug combinations are shown in Figure 6C in the main document. The nature of drug interaction (additive versus synergistic) was determined for each experiment by calculating combination index (CI) values using CalcuSyn software. Resulting fractional effects and CI values are shown. A CI value of 1 indicates an additive effect, whereas CI values below 1 indicate synergistic drug effects.

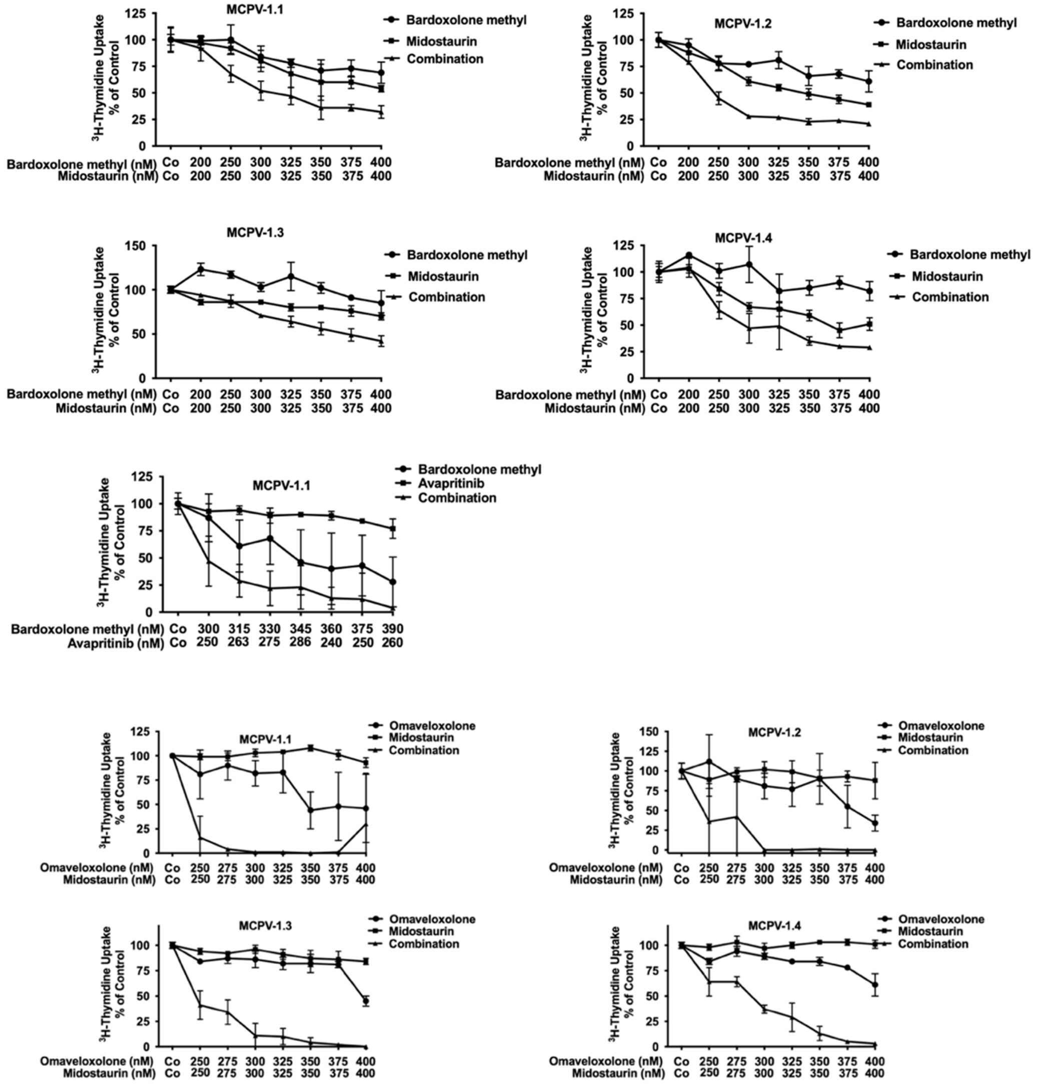

John et al, Supplementary Figure S6E

Bardoxolone methyl and omaveloxolone produce cooperative growth-inhibitory effects with midostaurin and avapritinib

MCPV-1 cells were incubated in control medium (Co) or medium containing either bardoxolone methyl, omaveloxolone or midostaurin/avapritinib alone, or a combination (at fixed ratio) of two drugs (at the concentrations indicated) for 48 hours. Then, proliferation was measured by assessing ^{3}H-thymidine uptake. Results show the percentage of ^{3}H–thymidine uptake compared with medium control and represent the mean±S.D. of triplicates from 1 typical experiment. At least two additional experiments with corresponding results were performed for all combinations shown.

Figure S7: Triple combinations of SMA-ZnPP and "synthetic triterpenoid + KIT TKI" in neoplastic MC

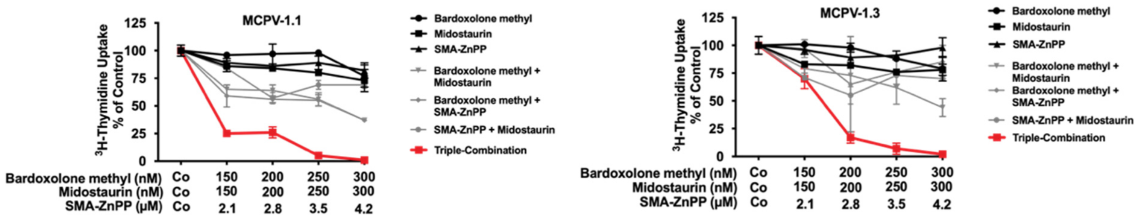

John et al, Supplementary Figure S7

SMA-ZnPP sensitizes neoplastic mast cells against the combination "synthetic triterpenoid + KIT TKI"

MCPV-1 cells were kept in control medium (Co) or in the presence of a single drug ((black lines: bardoxolone methyl/omaveloxolone (●-●); one of the TKI: midostaurin/avapritinib/nintedanib (■-■); SMA-ZnPP (▲-▲)), a two-drug combination ((grey lines: "Bardoxolone methyl/omaveloxolone + midostaurin/avapritinib/nintedanib" (▼-▼); "Bardoxolone methyl/omaveloxolone + SMA-ZnPP" (♦ - ♦); "SMA-ZnPP + midostaurin/avapritinib/nintedanib" (●-●) or the three-drug combination "bardoxolone methyl/omaveloxolone + midostaurin/avapritinib/nintedanib + SMA-ZnPP" (red line; □-□)) for 48 hours before ^{3}H-thymidine-uptake was evaluated. Results are calculated as percent of control and represent the mean±S.D. of triplicates of one representative experiment. At least two additional experiments with corresponding results were performed for all combinations shown.
